# A simple guide to *de novo* transcriptome assembly and annotation

**DOI:** 10.1093/bib/bbab563

**Published:** 2022-01-24

**Authors:** Venket Raghavan, Louis Kraft, Fantin Mesny, Linda Rigerte

**Affiliations:** Quantitative and Computational Biology, Max Planck Institute for Biophysical Chemistry, 37077 Göttingen, Germany; Quantitative and Computational Biology, Max Planck Institute for Biophysical Chemistry, 37077 Göttingen, Germany; Multitrophic plant-microbe interactions, Max Planck Institute for Plant Breeding Research, 50829 Köln, Germany; Multitrophic plant-microbe interactions, Max Planck Institute for Plant Breeding Research, 50829 Köln, Germany

**Keywords:** de novo, transcriptome, assembly, annotation, tools, RNA-seq

## Abstract

A transcriptome constructed from short-read RNA sequencing (RNA-seq) is an easily attainable proxy catalog of protein-coding genes when genome assembly is unnecessary, expensive or difficult. In the absence of a sequenced genome to guide the reconstruction process, the transcriptome must be assembled *de novo* using only the information available in the RNA-seq reads. Subsequently, the sequences must be annotated in order to identify sequence-intrinsic and evolutionary features in them (for example, protein-coding regions). Although straightforward at first glance, *de novo* transcriptome assembly and annotation can quickly prove to be challenging undertakings. In addition to familiarizing themselves with the conceptual and technical intricacies of the tasks at hand and the numerous pre- and post-processing steps involved, those interested must also grapple with an overwhelmingly large choice of tools. The lack of standardized workflows, fast pace of development of new tools and techniques and paucity of authoritative literature have served to exacerbate the difficulty of the task even further. Here, we present a comprehensive overview of *de novo* transcriptome assembly and annotation. We discuss the procedures involved, including pre- and post-processing steps, and present a compendium of corresponding tools.

## Introduction

Ribonucleic acids (RNAs) are an important class of biomolecules in cells and organisms. They represent the output of the genome being transcribed or expressed—the *transcriptome*. Numerous types of RNA exist, with each playing an important role in gene expression, and ultimately, in linking the genotype to the phenotype [1]. Ribosomal RNAs (rRNAs) and transfer RNAs (tRNAs), for instance, constitute the translation machinery that synthesizes proteins. The latter along with non-coding RNA (ncRNA) species also exert regulatory control over important biological processes [2, 3] including gene expression itself [4]. Messenger RNAs (mRNAs) constitute an important class of RNA. The sequences of mRNAs encode information that is used by the ribosomal machinery to synthesize proteins (translation). Hitherto non-mRNA species have been considered ‘non-coding’, assuming that they cannot be translated. However, this has been challenged by recent evidence indicating that regulatory long non-coding RNAs (lncRNAs) can in fact code for short peptides [5], underscoring the need for improving our understanding of these important molecules.

With the advent of affordable next-generation sequencing (NGS) platforms [6], high-throughput profiling of RNA using sequencing (RNA-seq) [7, 8] has become the preferred method of interrogating transcriptomes [7, 9]. RNA-seq can be used for a variety of purposes [7, 10]. The most popular use cases are establishing a catalog of an organism’s genes and proteins (transcriptome functional annotation) and studying changes in gene expression (differential expression analysis). ‘RNA-seq’ commonly refers to the so-called ‘bulk’ RNA-seq approach wherein material from a population of cells are pooled together for sequencing (e.g. all cells in a protozoan organism) as opposed to the increasingly popular single-cell RNA-seq (scRNA-seq) approach [11] wherein RNAs are isolated individually from single cells. We focus on the bulk RNA-seq approach in this paper.

Most RNA-seq studies today rely on short-read sequencing [7, 12, 13]. Here, the RNA molecules are isolated and enriched (usually for mRNA [7]), and reverse transcribed into complementary DNA (cDNA). The cDNA sequences are fragmented, randomly primed and amplified using PCR to yield an RNA-seq cDNA library which is then processed by the sequencing instrument [12, 14]. The sequencing output is in the form of millions of ‘short’ reads, which are sequences over an alphabet denoting a series of nucleotides (e.g. GATTACA). Such short-read sequences may be anywhere between 50 and 250 bp (base pairs) long; the library used for sequencing is often ‘sized’ (i.e. filtered) to retain only fragments of a certain length (e.g. 350 bp). The short reads must then be assembled into the sequences they originated from. This is the computationally challenging task of transcriptome assembly [15].

The sequences can be assembled either reference-guided or *de novo* [15]. The reference-guided approach requires the genome of the organism or a closely related species as an input. The reads can then be mapped to this ‘reference’ genome to determine which genes the reads originated from, and subsequently reconstruct the corresponding transcripts [15]. ***De novo* transcriptome assembly**, in contrast, is ‘reference-free’. The process is *de novo* (Latin for ‘from the beginning’) as there is no external information available to guide the reconstruction process. It must be accomplished using the information contained in the reads alone. This approach is useful when a genome is unavailable, or when a reference-guided assembly is undesirable. For instance, in opposition to a *de novo* assembler successfully producing a transcript, a reference-guided approach might not be able to reconstruct it correctly if it were to correspond to a region on the reference containing sequencing or assembly gaps [15, 16]. *De novo* assembly is discussed in detail in Section *De novo* transcriptome assembly. However, *de novo* assembled sequences are uninformative on their own. They must be assigned human-readable identifiers and have their functional and evolutionary properties characterized in order to have their biological relevance elucidated. This is the process of **transcriptome annotation**. As the objective of the procedure is to elucidate the functions of the sequences, it is also often referred to as ‘functional’ annotation.

Short-read RNA-seq is affordable, easily accessible and has low error rates. And importantly, it has a large community of established practitioners, literature, tools and other resources. As a result, the popularity of the approach continues to proliferate across the biological sciences. It has become especially popular for studying non-model organisms (for example, in the ecological sciences [17]), as a *de novo* transcriptome is an acceptable substitute for an absent genome. For example, it has been used to study zooplankton [18], bats [19], fruits [20] and pathogens [21]. There is now also considerable interest in ‘in-housing’ the *in silico* assembly and annotation workflows as the required computational resources have become easily accessible [22, 23]. It is now possible to sequence, assemble *de novo* and annotate a transcriptome within the confines of one’s own laboratory. However, the path to an annotated, *de novo* assembled transcriptome can be challenging. Those interested must not only acquaint themselves with the procedures involved, but also select the right set of tools for this purpose. These issues are non-trivial, and can become overwhelming. RNA-seq literature reveals many variations on the same theme, with a variety of tools and combinations of processing steps having been used. Furthermore, RNA-seq is a computationally intensive task. Becoming acquainted with the computational resources necessary can also be a hurdle.

**Figure 1:**
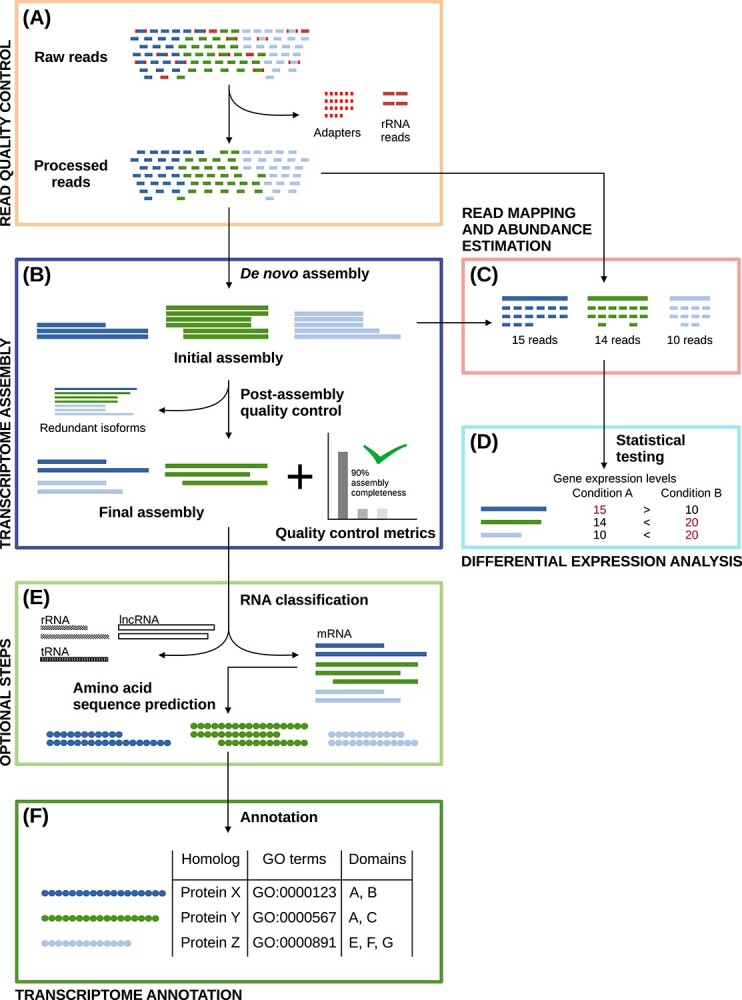
Assembly and annotation workflow. **(A)** Quality control of the raw reads by filtering for erroneous reads and sequencing artifacts. **(B)** Sequence assembly including clustering into groups of isoforms and removing redundant sequences (isoforms are transcript variants arising from alternative splicing). **(C)** Mapping the raw reads to the assembled sequences for either quality control of the assembly or for differential expression analysis. **(D)** Applying statistical tests for identification of changes in expression levels. **(E)** Classifying sequences by RNA species and translating into protein sequences before annotation. **(F)** Annotating sequences on the basis of sequence similarity, identifying sequence features (such as functional domains) and annotating Gene Ontology terms.

Here, we present a step-by-step overview of the *de novo* transcriptome assembly and annotation workflow (Figure 1). In brief, the RNA-seq data must first be quality controlled (Figure 1 panel (A), Section ‘Pre-assembly quality control and filtering’). For instance, this can include excluding reads originating from rRNAs, and removing adapter sequences. Subsequently, the data can be assembled *de novo* to obtain the transcriptome, whereafter they must be quality controlled once again in order to produce a final assembly free of assembly artifacts (Figure 1 panel (B), Sections ‘*De novo* transcriptome assembly’, ‘Post-assembly quality control’, ‘Alignment and abundance estimation’ and ‘Assembly thinning and redundancy reduction’). Read alignment and transcript abundance estimation (Figure 1 panel (C), Section ‘Alignment and abundance estimation’) are performed both as quality control measures, and to estimate gene/transcript expression levels for differential expression analysis (Figure 1 panel (D), Section ‘Differential expression analysis’). If the RNA-seq data are suspected to contain non-mRNA species, RNA classification can be carried out to classify and filter the data (Figure 1 panel (E), Section ‘RNA classification’). Protein sequences are useful in many contexts (including annotation), and therefore, the transcriptomic sequences can be translated into their amino acid counterparts (Figure 1 panel (E), Section ‘Sequence translation’). Finally, the nucleotide (and/or translated protein) sequences can be annotated to assign human-readable identifiers to them, and elucidate their biological roles (Figure 1 panel (F), Section ‘Transcriptome functional annotation’).

In the subsequent sections, alongside a brief conceptual introduction of each procedure, we present a compendium of the relevant state-of-the-art-tools. As transcriptome annotation is not well-addressed in literature, we have discussed this procedure in detail. Transcriptome annotation involves a myriad of processes which we present and discuss as independent, compartmentalized steps. We also discuss a number of transcriptome annotation pipelines that automate the entire procedure (Section ‘Transcriptome annotation suites’). The need may arise to compare multiple transcriptomes, for instance to infer conserved orthologs [24]. We have discussed comparison of transcriptomes and relevant tools in the Section ‘Comparing transcriptome assemblies’. *De novo* assembly and annotation workflows continue to grow in complexity, both in terms of the number of tools used and samples processed. Therefore, automated workflows are needed to make the procedures tractable, scalable and reproducible. To this end, we have devoted an entire section to the important topic of bioinformatic workflow managers which can be used to construct and orchestrate such workflows (Section ‘Workflow managers’). For the interested newcomer to the field, we briefly summarize some of the computational prerequisites to be aware of in Section ‘Computational and programmatic considerations’. Finally, it can potentially be unclear as to what one should annotate in a *de novo* transcriptome, and where these annotations can be published. We address these issues in the final section of this document (Section ‘What to annotate and where to publish’). As we name and discuss well over 100 different tools in this paper, we have also supplied a spreadsheet summarizing these as a supplement (Table S2). Our hope is that this publication can serve as a primer to the topic, and as a ‘directory’ of procedures, tools and literature that users can consult and use in pursuit of the perfect *de novo* assembled transcriptome.

## Pre-assembly quality control and filtering

**Figure 2:**
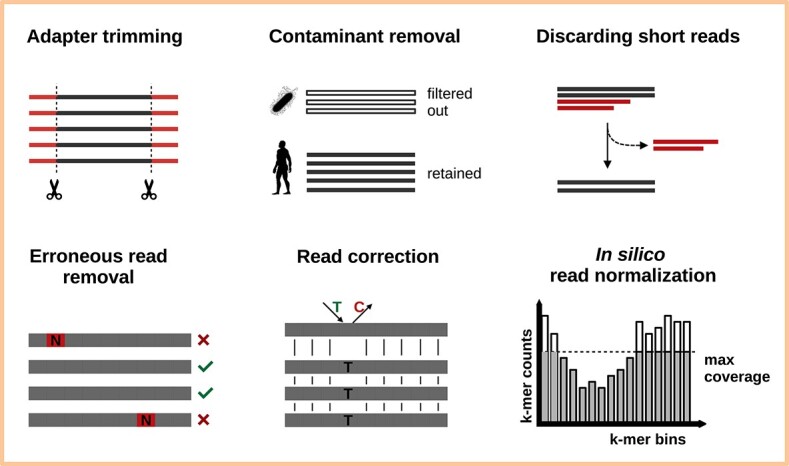
Short-read quality control and data cleansing involve procedures such as adapter trimming, removing short reads and erroneous reads containing N-bases, read correction by comparison to other reads, and excluding reads originating from contaminant sources (e.g. pathogens in a host species). *In silico* read normalization can be a useful pre-processing step for very large data sets (>200M reads) where it can significantly improve assembler performance by selectively reducing the reads in a manner such that the transcriptomic complexity of the original data set is retained.

The reads generated by the sequencer constitute the data underpinning the assembly. While modern sequencers have low error rates, the data they produce are not error-free [25]. Properties of the reads including their abundance, read length, stranded-ness, paired-ness, overall GC content, k-mer composition and embedded errors directly affect the quality of the assembly, and by extension all subsequent procedures [26]. Therefore, the first step in *de novo* transcriptome assembly involves quality controlling the raw read data (Figure 2 highlights some such procedures). Quality control here implies both inspection of the data, and subsequent correction or filtering if considered necessary.

The short-read sequence inspection tool FastQC can be deployed as the first step of the pre-assembly quality control process. The tool provides a summarized overview of read quality metrics such as per-base PHRED quality scores, average incidence of ‘N’ (i.e. undefined) bases, GC content, read length distributions, identities of overrepresented sequences and presence of adapter sequences. A brief perusal of the report should indicate the measures that need to be taken. For instance, adapter sequences present in the reads may have to be removed, and the reads may perhaps have to be screened for contamination from non-target species. A recent alternative to FastQC is Falco [27], which can perform many of the same functions as FastQC. If multiple read data sets are being handled together, the bioinformatics report aggregator MultiQC [28] can be used to simultaneously inspect reports from not only FastQC but also numerous other tools (see https://multiqc.info/#supported-tools).

Subsequently, several measures can be applied to either correct or exclude aberrant reads. The first such procedure that can be applied is k-mer based read error correction using the tool Rcorrector [29]. This can be used to fix random errors generated during sequencing. However, such errors can be indistinguishable from single nucleotide polymorphisms (SNPs), and can lead to sequence variants being lost from the assembly.

If quality control metrics indicate the presence of adapter sequences in the data, these should be removed prior to assembly. Although adapter removal may have been performed by the sequencing facility, it is a good practice to scan for and eliminate residual adapters all the same. If only adapter trimming is desired, the dedicated trimming software cutadapt [30] is a good option as it is capable of error-tolerant adapter detection. The tool TrimGalore is a wrapper built around cutadapt and FastQC featuring some added functionality such as length-based sequence filtering which can be useful for discarding extremely short reads resulting from adapter removal. Finally, BBDuk from the BBTools [31] suite can also be used for the purpose of adapter removal. All three tools accept user-defined adapter sequences. BBDuk includes a set of common adapters and contaminants such as vectors. Therefore, explicit user input is not required in most cases.

It is often insufficient to perform only adapter removal. For instance, sequencing data often include reads containing ambiguous base calls (identified via the character N in the sequence). Retaining such sequences only serves to confound the assembly and downstream analyses, as the exact nucleotide at that position in the read cannot be ascertained. Similarly, the data may also be filtered to retain only those reads (or portions thereof) containing bases with a certain minimum quality (Q) score. These quality scores [32] encode the probability of that particular base-call being wrong; for instance, a base with a Q value of 30 has a 0.001% chance of being erroneous. Reads carrying some maximum number of low-quality base calls can either be discarded entirely, or trimmed if the bases occur on the flanks. Likewise, it may be beneficial to discard reads that are extremely short (e.g. ~30 nt). Although these steps can be performed by user-written scripts, it is more efficient to carry them out using purpose-built tools. One such all-in-one tool for NGS read quality control is fastp [33]. It can perform a wide variety of read quality control procedures including (but not limited to) automated adapter detection and removal, N-containing read removal, low-quality base filtering, overlap-based read correction (with paired-end reads), paired-end read merging and poly-X read trimming. An equivalent alternative is the tool Trimmomatic [34] which shares many of its features.

Once basic cleaning has been performed, the data can be assessed for the presence of contaminants. These are typically reads that do not originate from the organism and/or RNA species of interest. Contaminants can be broadly classified into two categories: foreign sequences and cognate contaminants. As the name suggests, foreign contaminants are reads belonging to off-target species (for instance, reads originating from an endosymbiont bacterium in an eukaryote organism of interest). Foreign contaminants can be detected—and optionally removed—using a short-read taxonomic classifier. kraken2 [35] is a fast short-read taxonomic classifier intended for metagenomic analysis. In the RNA-seq context, it can be used to classify and remove all reads not originating from the taxon of interest. For instance, with a eukaryotic read dataset, kraken2 could be used to exclude reads classified as bacterial, archaeal, fungal or from plants. kraken2 offers ready-made reference sequence databases for classification; these can be found at https://benlangmead.github.io/aws-indexes/k2. An alternative to kraken2 is Centrifuge [36] which can perform the same classifications, but with a smaller memory footprint. FastQ Screen is a screen-only alternative that can detect—but not remove—contaminants based on a user-supplied database.

In contrast, cognate contaminants are reads originating from off-target RNA species. For instance, although most RNA-seq methods select for mRNA sequences, it is still possible for off-target species to get represented in the data set in sizable quantities. This is especially true for rRNA sequences[37–39]. Reads originating from rRNAs are best detected and removed using SortMeRNA [40]. This tool was originally designed to filter out rRNA reads from metatranscriptomic data, but it can also be used with RNA-seq data. The tool maps inputs against custom rRNA databases (derived from Rfam [41] and SILVA [42]) to classify them as rRNA or non-rRNA reads. This is useful for enriching the data for reads from coding sequences prior to assembly. Other cognate contaminants such as long non-coding RNAs (lncRNAs) are best detected and dealt with post-assembly. This has been discussed further in the Section ’RNA classification’.

Modern RNA-seq studies now routinely sequence hundreds of millions of reads with the objective of reconstructing all expressed transcripts to full length to construct so-called ‘reference’ transcriptomes. Although this enhances sensitivity for recovery of lowly expressed transcripts [43, 44], it also has the side effect of producing a large number of reads for transcripts that are already well represented with significantly fewer total reads. Such an overabundance of reads (for well-represented transcripts) can quickly lead to unacceptable assembler performance and very long runtimes. This typically appears to occur at read depths exceeding 200 million reads [45]. In such situations, performing *in silico* normalization on the reads prior to assembly can significantly alleviate the aforementioned performance issues. Here, reads are quantified on the basis of their k-mer abundances, and are either retained or rejected based on user-defined thresholds [45]. The outcome is a strong reduction of the read volume in such a manner that full length reconstruction of a large majority of the transcript cohort can be achieved despite fewer reads being input to the assembler [45, 46]. Some tools that can perform *in silico* read normalization include khmer (using the diginorm algorithm) [47], Bignorm [48], NeatFreq [49] and ORNA [50]. The Trinity [46] assembler also offers in-built *in silico* normalization [45, 46].

**Links:**

BBTools - https://sourceforge.net/projects/bbmap/, https://jgi.doe.gov/data-and-tools/bbtools/

Bignorm - https://git.informatik.uni-kiel.de/axw/Bignorm

Centrifuge - https://github.com/DaehwanKimLab/centrifuge

cutadapt - https://github.com/marcelm/cutadapt

Falco - https://github.com/smithlabcode/falco

fastp - https://github.com/OpenGene/fastp

FastQC - https://www.bioinformatics.babraham.ac.uk/projects/fastqc/

FastQ Screen - https://www.bioinformatics.babraham.ac.uk/projects/fastq_screen/

khmer - https://github.com/dib-lab/khmer

Kraken2 - https://github.com/DerrickWood/kraken2

MultiQC - https://multiqc.info

NeatFreq - https://github.com/bioh4x/NeatFreq

ORNA - https://github.com/SchulzLab/ORNA

rCorrector - https://github.com/mourisl/Rcorrector

SortMeRNA - https://github.com/biocore/sortmerna

TrimGalore - https://github.com/FelixKrueger/TrimGalore, https://www.bioinformatics.babraham.ac.uk/projects/trim_galore/

Trimmomatic - https://github.com/usadellab/Trimmomatic

## *De novo* transcriptome assembly

RNA-seq reads contain a mixture of fragments corresponding to different parts of different transcripts. Transcriptional noise [51, 52], sequencing artifacts [53] and transcript isoforms originating from alternative splicing [54, 55] are also represented in these data. The objective of assembly is to accurately disambiguate the origin of the reads and reconstruct an accurate representation of the parent sequences. This is typically achieved by examining overlaps between reads (or subsequences thereof) in order to concatenate them into longer contiguous sequences (contigs) [15, 56].

**Figure 3:**
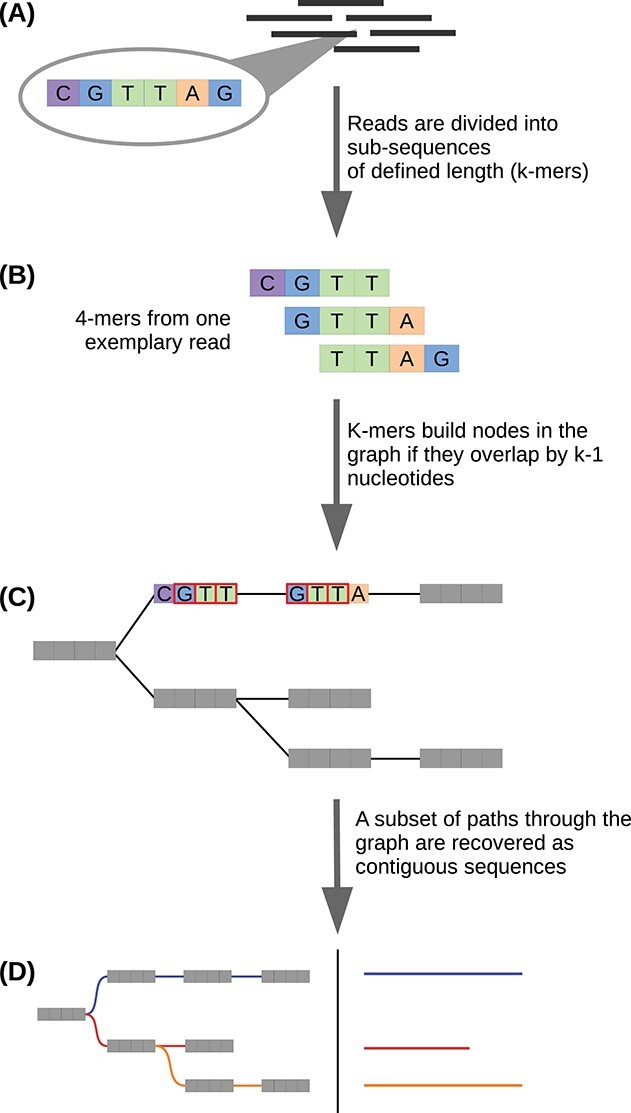
A typical graph-based approach to *de novo* transcriptome assembly. The basic idea is to establish a catalog of sub-strings from the RNA-seq reads, and compose these into a graph (or set of graphs) wherein the sub-strings are connected if an overlap between them exists. This establishes paths through the graph(s) which correspond to the transcripts the reads (potentially) originated from. **(A)** Short nucleotide reads 50–250 nt in length are the inputs for the assembly process. If paired-end reads are supplied, the respective mates are merged into a single contiguous read prior to assembly. Highlighted here is a 6 nt portion of a single read (CGTTAG). **(B)** For each read, all possible sub-sequences of length k are generated (k-mers). The 4-mers (k = 4) originating from the 6 nt nucleotide fragment from the previous step are indicated here as examples. **(C)** Subsequently, each k-mer becomes a node (also called vertex) in the graph, and an edge is established between any two nodes that share a k-1 nucleotide overlap with each other. Edges are established between any two nodes that satisfy this overlap requirement. As a simplified example, an edge connecting the first and second 4-mers from the previous step is highlighted here as existing as a part of a De Brujin graph. **(D)** Finally, different paths through the graph(s) are traversed and recovered as independent sequences. Not all paths through the graph are recovered; the subset of paths that represent valid transcripts is determined algorithmically.

Assembly algorithms are mostly based on using k-mers as assembly units instead of whole reads. A *k-mer* is a sub-string of length k derived from a particular read [46]. The first step in the assembly process is to construct a dictionary of all possible k-mers (for a given k) and the reads these k-mers originate from. Most modern assemblers are graph-based in that they represent the *k-mers* as nodes in a so-called De Bruijn graph (Figure 3). Subsequently a contig is a path through the graph, where each distinct k-mer represents a vertex in the graph. Edges are formed between two k-mer vertices if they have an overlap of exactly k-1 nucleotides. In this way paths through the graph correspond to possible sequences the k-mers originated from (Figure 3). Paths are extended until no further overlap-based extensions are possible [46]. Then each possible path through the graph is traversed and recovered as a separate contig corresponding to a single transcript.

*De novo* assembly does not produce a single, large De Bruijn graph like in a genome assembly but instead many disconnected subgraphs that, when disentangled, correspond to groups of related sequences (transcript isoforms or very closely related paralogs). Subsequently, post-processing steps may be implemented to filter and group contigs to yield a representative set of the assembled sequences. De Bruijn graph-based assembly is sensitive to the choice of k-mer length as it dictates the set of contigs assembled by controlling the complexity of the graphs. Generally speaking, shorter k-mer lengths imply a higher chance of error-free k-1 overlap between any two k-mers. As such, shorter k-mer lengths contribute to the recovery of lowly expressed transcripts, but also lead to a larger number of false positive (incorrect/non-existent) contigs being assembled by connecting k-mers from unrelated reads [56, 57]. On the other hand, choosing a longer k-mer length would reduce the total number of contigs assembled, but also suppress the recovery of lowly expressed transcripts as fewer reads would be able to satisfy the k-1 overlap requirement in an error-free manner. Therefore, the choice of the k-mer length defines a major trade-off in the assembly process [56]. Assembly tools usually supply a default value (or range thereof) for *k* which can be modified by the user.

A large number of tools are available for *de novo* assembly, and choosing one is a critical step in the workflow. The most prominent De Bruijn graph-based assembler is Trinity [45, 46]. Since its release in 2011, the corresponding publication [46] has been cited over 10 000 times. It is robust and easy to use with an extensive set of associated tools, and a large user community. Most importantly, its general performance is consistently high and on par with other novel assemblers [56, 58], making it a trustworthy choice for assembly. A salient feature of Trinity is that it identifies sets of contigs that may be biologically related to one another (e.g. splice variants [59, 60]), and designates these as gene isoforms. This feature is especially useful for differential gene expression analysis with *de novo* assembled data, where it is common practice to aggregate the expression of related transcript isoforms into that of a representative ‘gene’, as this is considered to be robust [61, 62]. There are numerous other equally capable *de novo* assemblers [58]. A non-exhaustive list includes SOAPdenovo-Trans [63], Oases [64], Trans-ABySS [65], IDBA-Tran [66], inGAP-CDG [66], RNA-Bloom [67] and rnaSPAdes [56]. All of these tools except for SOAPdenovo-Trans apply a multiple k-mer strategy, aiming to make use of the advantages of small and large k-mer lengths to maximize transcript recovery. RNA-Bloom is actually specialized toward assembling single-cell RNA-seq but can also assemble bulk RNA-seq. In terms of performance and assembly output, rnaSPAdes and Trans-ABySS are similar to Trinity [58].

Splicing graph assemblers are a variant of De Bruijn graph assemblers. In this approach, each vertex corresponds to an exon, while the edges represent splice junctions [68, 69]. The paths through the graphs correspond to transcript isoforms. The graphs generated are less entangled in comparison to a traditional De Bruijn graph [70]. Representatives assemblers from this category include Bridger [71], BinPacker [57], TransLig [72], DTA-SiST [68] and IsoTree [70].

Choosing an assembler can be a difficult task. *Holzer et al.* [58] recently concluded in a broad evaluation of common transcriptome assemblers—using a variety of data sets from different species—that assembler performance is very dependent on the data supplied to it. As a consequence, they were unable to declare an unanimous ‘best’ assembler. We concur, and recommend comparing at least two different assemblers and multiple k-mer lengths.

**Links:**

BinPacker - https://github.com/macmanes-lab/BINPACKER

Bridger - https://github.com/fmaguire/Bridger_Assembler

inGAP-CDG - https://sourceforge.net/projects/ingap-cdg/

DTA-SiST - https://github.com/jzbio/DTA-SiST

IDBA-tran - https://github.com/loneknightpy/idba

IsoTree - https://github.com/david-cortes/isotree

Oases - https://github.com/dzerbino/oases

RNA-Bloom - https://github.com/bcgsc/RNA-Bloom

rnaSPAdes - https://github.com/ablab/spades

SOAPdenovo-Trans - https://github.com/aquaskyline/SOAPdenovo-Trans

Trans-ABySS - https://github.com/bcgsc/transabyss

TransLig - https://sourceforge.net/projects/transcriptomeassembly/

Trinity - https://github.com/trinityrnaseq/trinityrnaseq

## Post-assembly quality control

Due to the noisy nature of RNA-seq data, *de novo* assemblies can contain intronic sequences and other ‘transcriptional’ byproducts. Further, the assembly process itself is not error-free [61]. For instance, unrelated but highly similar transcripts may be incorrectly fused together into a single contig during the assembly process (i.e. a chimera [73]). Or the choice of k-mer length might have been inappropriate, leading to a highly fragmented assembly wherein multiple contigs together would yield a longer, complete sequence (that might have been otherwise assembled with a different choice of k-mer length). Therefore, assessing the quality of a *de novo* transcriptome assembly is a crucial step before annotation and other downstream procedures. A low-quality assembly can lead to erroneous interpretations in a variety of scenarios including gene identification and differential expression analysis.

The quality of an assembly can be assessed from several perspectives. First is sequence length and fragmentation. An assembly with many short contigs can be considered fragmented. It is possible that this is the result of improper assembly or poor sequencing. Tools such as SeqKit [74] can be used to calculate sequence length statistics (such as the N50 value) that are helpful in this regard. Second is read support—the fraction of all reads that map back to the assembly. A good quality assembly will have made use of most of the reads that went into it. Further, the proportion of reads that map to multiple sequences would be low (but this cannot be guaranteed, as a gene may genuinely have many transcript isoforms). All of these metrics can be checked easily by aligning the reads against the assembled sequences. Read support and alignment estimation tools are discussed in Section ‘Alignment and abundance estimation’. The GitHub Wiki of the Trinity  *de novo* assembler https://github.com/trinityrnaseq/trinityrnaseq/wiki lists several other methods to assess the quality of an assembly including interrogating the strand-specificity of the assembly in case of prior strand-specific sequencing, and calculating the ExN50 statistic [58, 75].

At this juncture, we would like to take a moment to caution readers with regards to the application of the N50 statistic to transcriptome assemblies. The N50 is a simple metric which describes the sequence length at which half the nucleotides in the genome assembly are in sequences equal in or longer than this length [76]. The goal would then be to maximize the N50 value as this would indicate complete assembly of all genomic elements (e.g. multiple chromosomes). This is inappropriate for transcriptome assemblies as the objective is recovery of many (relatively) short full-length sequences, and not the construction of a few very long contigs. Given the presence of transcript isoforms, short contigs resultant from transcripts with low coverage, and overly long contigs resultant from overzealous assembly of multiple isoforms, the N50 statistic can become heavily skewed, thereby presenting a biased overview of the assembly. The ExN50 metric is a modification to the traditional N50 making it suitable for assessing transcriptome assemblies. Here, the N50 value is calculated only for the top X% of the cumulative expression levels. The length reported as corresponding to ExN50 is a ‘gene’ length obtained as the expression-weighted sum of the corresponding isoform lengths. This approach neatly sidesteps the issues posed by the plurality of short, lowly expressed transcripts and long isoforms from highly expressed transcripts, as these are now gathered into representative genes. This metric is currently only implemented for the Trinity assembler.

An alternative approach to checking the quality of the assembly is to assess its composition. A good quality assembly would ideally have recovered a large fraction of the transcriptome that had been sequenced. The most popular method in this regard is to test the assembly for the presence of orthologs to certain genes that are universal, persistently expressed and occur almost exclusively as single copies in the genome. If a transcriptome has been properly sequenced and assembled, orthologs to a large majority of these should be found. This analysis can be performed using the tool BUSCO (Benchmarking Universal Single-Copy Orthologs) [77]. The tool maintains curated sets of universal single-copy genes from OrthoDB [78]. The ‘completeness’ of the assembly is assessed by how many of the universal genes have matches in the input data and whether these matches are duplicated, fragmented or full length. As a general rule a good quality assembly should have fairly high BUSCO completeness scores: $>80\%$  BUSCO genes should have matches in the transcriptome, and very few matches should be missing or fragmented. If an assembly has a high proportion of missing and fragmented BUSCO genes, this is indicative of poor quality. In general, *de novo* transcriptome assemblies will have many duplicate matches to the BUSCO sequences. This is caused by the presence of closely related transcripts that represent splicing isoforms, and thus is not necessarily indicative of unwanted redundancy in the assembly. The issue of redundancy is discussed in detail in Section ‘Assembly thinning and redundancy reduction’. As the tool was originally designed for genomic assemblies, BUSCO does not account for this phenomenon. An alternative to BUSCO is the domain-based quality assessment tool DOGMA [79]. In this case, instead of scoring on the basis of conserved genes, completeness is instead assessed on the basis of conserved protein domains.

In a similar vein, the assembly quality can also be checked on the basis of the provenance of the assembled sequences. Ideally, for a given organism, a vast majority of the assembled transcriptome sequences should map to its own sequences in an external database (e.g. from genomic sequencing; or those from closely related species). Concomitantly, sequences that do not map in this manner (or map to off-target organisms) can be considered contaminants and filtered out, yielding an improved assembly. But this may potentially discard novel, un-annotated sequences, so it must be done with caution. There are several popular sequence search/alignment tools and sequence databases that can be used for checking the provenance of the assembled sequences. These are discussed in Section ‘Identity assignment via homology transfer’. If the sequencing reads have been processed prior to assembly (as discussed in Section Pre-assembly quality control and filtering), this quality control may not be as useful.

Several integrative tools have been developed over the years with an eye on assessing the quality of *de novo* transcriptome assemblies. These tools generally expand upon the basic read mapping metrics mentioned above and calculate additional statistics. They may also offer the option to run BUSCO and other tools internally, compare two or more versions of an assembly and compare the assembled sequences against a genome or a database of known sequences (as an example, see metrics indicated in this website). The most popular tool in this regard is TransRate [80] which incorporates many of the metrics mentioned above. The tool also checks for the presence of chimeric sequences, whose removal generally improves transcriptome assemblies [56, 81]. DETONATE [82] and rnaQUAST [83] are tools developed in the same vein as TransRate, but only rnaQUAST is still being developed (as of this publication). TransRate and DETONATE, however, appear to continue to be in use judging from recent citations (for instance, see supplementary materials from *Ceschin et al.* [84]). A recent development is the Bellerophon pipeline [85], which offers a comprehensive quality assessment and filtration tool that integrates several tools including TransRate, the clustering suite CD-HIT [86] and BUSCO. In addition to assessing quality, the tool also automatically applies measures (such as filtering out very lowly expressed transcripts) to improve the quality of the assembly. The only inputs required are the assembly and the reads.

Quality controlling a *de novo* assembly can require multiple rounds of assembly (for instance to test different k-mer lengths), which can quickly become a tedious undertaking. There are several tools that encapsulate pre-processing, assembly, quality control measures and even annotation together (often using bioinformatic workflow managers; see Section ‘Workflow managers’) to enable turnkey production of high-quality transcriptomes. Some of the popular tools in this regard include DRAP [87], EvidentialGene and the multi-assembler approach-based pipelines Oyster River Protocol [88], TransPi [89] and Pincho [90].

**Links:**

Bellerophon Pipeline - https://github.com/JesseKerkvliet/Bellerophon

BUSCO - https://busco.ezlab.org/

DETONATE - https://github.com/deweylab/detonate

DOGMA - https://domainworld-services.uni-muenster.de/dogma/ (web server), https://ebbgit.uni-muenster.de/domainWorld/DOGMA (source code)

DRAP - http://www.sigenae.org/drap/

EvidentialGene - http://arthropods.eugenes.org/EvidentialGene/

The Oyster River Protocol - https://oyster-river-protocol.readthedocs.io/en/latest/index.html

Pincho - https://github.com/RandyOrtiz/Pincho

rnaQUAST - https://github.com/ablab/rnaquast

TransRate - https://github.com/blahah/transrate

and http://hibberdlab.com/transrate/

SeqKit - https://github.com/shenwei356/seqkit

TransPi - https://github.com/palmuc/TransPi

Trinity Wiki - https://github.com/trinityrnaseq/trinityrnaseq/wiki

## Alignment and abundance estimation

Read alignment and transcript abundance estimation are typically used for differential expression analysis in the broader context of RNA-seq. Read mapping is a pre-requisite for abundance estimation [91]. However, alignment metrics can also be used to quality control the assembly. A ‘good quality’ *de novo* assembled transcriptome would have a large majority of the reads mapping/aligning to the assembly, i.e. most reads will have had been used in its construction. This is assessed as the ‘read support’ for the assembly. As a thumb rule, a good assembly would have $>80\%$ read support, and would have a low proportion of un-mapped reads. Reads can also map to more than one contig (multi-mapping reads). This can occur, for instance, when the assembly contains transcript isoforms that share exons. (Multi-mapping reads are discussed also in Section ‘Assembly thinning and redundancy reduction’.)

Abundance estimation, as the name implies, refers to the process of inferring the expression level of the transcripts in the assembly. Abundances are *estimated* because it is impossible to disambiguate the source of multi-mapping reads, and the true expression levels of transcripts are usually unknown. As such, most techniques typically produce maximum likelihood values for transcript abundances. These values which include read support (on a per-transcript basis) and a normalized expression metric such as transcript per million (TPM) [91]. These values are crucial for differential expression analysis (see Section ‘Differential expression analysis’), but can also be used for assembly quality control purposes. For instance, transcripts of questionable biological significance typically have low expression levels, and can be filtered out from the assembly based on their TPM metrics. TPM calculations can be easily performed using a dedicated tool such as TPMCalculator [92].

Read alignment and abundance estimation can usually be done together. There are two main approaches to the combined procedure. The first is a two-step process where the reads are first aligned to the assembled contigs using a general purpose aligner such as Bowtie2 [93] or STAR [94]. The output is typically a BAM file which lists the sequences and the reads aligned to them (*Li et al.* [95] and http://www.htslib.org). This is then fed to a tool such as RSEM [96] (RNA-seq by Expectation-Maximization) to obtain abundance estimates. The alternative is a single-step approach known as pseudoalignment. Read alignment is computationally expensive as every nucleotide from the reads and assembled contigs must be compared. Pseudoalignment eschews this in favor of establishing the association between reads and contigs on the basis of k-mer similarities between them. There are two popular pseudoalignment tools, namely Kallisto [97] and Salmon [98]. Both tools are based on very similar approaches.

We would recommend using one of the pseudoalignment tools as opposed to the alignment-estimation workflow due to their speed [99], comparably high accuracy [100–102] and ease of use. Additionally, these tools can also run under an alignment-based mode if necessary, making them the versatile choice.

**Links:**

Bowtie2 - https://github.com/BenLangmead/bowtie2

Kallisto - https://github.com/pachterlab/kallisto

RSEM - https://github.com/deweylab/RSEM

Salmon - https://github.com/COMBINE-lab/salmon

STAR - https://github.com/alexdobin/STAR

TPMCalculator - https://github.com/ncbi/TPMCalculator

## Assembly thinning and redundancy reduction

*De novo* transcriptome assemblers typically produce many more sequences than would be expected based on number genes in the genome. For example, *Bryant et al.*[75] report having assembled over 1.5 million sequences for a transcriptome of the axolotl (*Ambystoma mexicanum*). The genome, in comparison, has ca. 23 000 genes [103]. The discrepancy between the number of genes and the number of transcripts assembled *de novo* boils down to the perception that transcription is a noisy, pervasive process. For instance, *Dunham et al.* [104] state that over $80\%$ of the *Homo sapiens* genome gets transcribed even though less than $3\%$ [105] of the transcribed products code for proteins. As such, the *de novo* assembled contigs include transcriptional artifacts, pre-mRNA and ncRNA in addition to the protein-coding transcripts [61]. Another source of extra sequences is alternative splicing [59, 60, 106] which manifests as transcript isoforms. It may not always be necessary to retain all such sequences. Assembly thinning can therefore be an important step toward obtaining a sequence set of a manageable size.

A straightforward approach to thinning is to manually select contigs that can be considered representative with respect to the entire assembly. This is infeasible unless the relationships between the assembled contigs is known *a priori*. Luckily, most popular assemblers classify the transcripts into groups of isoforms automatically. A representative isoform can be chosen in several different ways: the isoform with the highest read support, the longest isoform, or the isoform that produces the longest translated amino acid sequence, or even the isoform whose coding sequence (CDS) has the highest read support. All of these approaches may be equally effective, and are likely to be data set-dependent. It is recommended to choose a method based on the BUSCO scores and other quality metrics.

Should the gene-isoform relationship be unavailable, a simple approach to thinning would be to exclude transcripts that can be considered as being lowly expressed on the basis of abundance metrics such as TPM. These metrics can be calculated easily using one of the tools mentioned in the Section ‘Alignment and abundance estimation’. Thereafter, contigs with read support below some threshold (e.g. TPM  $< 1.00$) could be discarded from the assembly.

A more rigorous approach for assembly thinning is to use a clustering tool. This is especially useful in cases where the assembled contigs do not have the gene–isoform relationship disambiguated or the assembly is genuinely redundant (i.e. many contigs with nearly identical sequence have been assembled). The clustering tools CD-HIT [86, 107] and MMSeqs2 [108–111] use a combination of sequence identity and sequence coverage thresholds to group sequences together into clusters and extract representative sequences. The representatives are typically either the longest sequence in each cluster or the sequence with the most commonality with the cluster members. There are also several tools that have been developed specifically with *de novo* transcriptome assemblies in mind. Many of these tools work on the premise that shared read support—i.e. high proportions of multi-mapping reads within a set of reads corresponding to a set of transcripts—can be used to cluster the sequences together. Tools in this category include Corset [62], Grouper [112] and Compacta [113].

An interesting approach to assembly thinning is presented by the SuperTranscripts [114] tool. Instead of choosing a representative isoform for each gene cluster, the tool simply stitches all unique exons from the isoforms into a single, linear sequence. This ‘transcript-hybrid’ does not necessarily exist in a real biological context, but can nevertheless be useful. Super transcripts have great potential not only for analysis, e.g. for studying differential transcript usage, but also for assembly thinning without any sequence information loss. Assembly thinning is not the main objective but rather a side-effect.

It is important to note that assembly thinning should be performed only if absolutely necessary. The provenance of *de novo* assembled contigs are unknown, and they all therefore can carry significant biological information. Assembly thinning is an inherently heuristic task. It is entirely possible, for instance, to tune the parameters such that closely related paralogs get clustered together. In such an event, sequences that should be represented in the assembly will be lost. Further, in the case of isoforms, it is often impossible to identify a single best isoform [45]. For instance, the longest isoform is not necessarily the most expressed (and vice versa). It is not even necessary that the longest or the most expressed isoform is the one that is actually representative of the gene and the concomitant protein. The longest isoform may be the result of the assembler erroneously overextending the biologically relevant contig, or the result of an intron being retained in the transcript. Subsequently, the corresponding protein may not be the longest protein in the cohort, or may even be absent as a result of the corresponding ORF being aberrant. As such, extreme caution must be exercised when performing assembly thinning and redundancy reduction, as irreverent thinning can result in the loss of otherwise informative sequences from downstream analyses.

**Links:**

CD-HIT - http://weizhongli-lab.org/cd-hit/

Corset - https://github.com/Oshlack/Corset

Compacta - https://github.com/bioCompU/Compacta

Grouper - https://github.com/COMBINE-lab/grouper

MMseqs2 - https://github.com/soedinglab/MMseqs2

SuperTranscripts - https://github.com/Oshlack/Lace

## Differential expression analysis

Assessing changes in gene expression in response to changes in physiological or environmental conditions is one of the main objectives of the RNA-seq approach. In the simplest case, this is achieved by capturing the RNA from independent samples (in replicate) exposed to experimental and control conditions. Thereafter, the sequenced reads can be mapped to the organism’s genes to assess how differently the genes are expressed under the experimental circumstances as opposed to the control scenario. This is known as **differential expression (DE) analysis** [115]. Through such comparisons of expression, it is possible to obtain an understanding of the activity of genes under various circumstances.

In order to perform a DE analysis, a collection of gene sequences from the organism is required. The genes themselves can be used if an annotated genome is available. If no genome is available, a *de novo* assembled transcriptome can be used, with the transcripts acting as proxies for the genes. In a *de novo* transcriptome assembly for a DE analysis, the reads from all conditions and all replicates are pooled together for assembly: this produces a single, common ‘reference’ transcriptome against which the reads can then be mapped and quantified.

Subsequently, the data can be analyzed for indications of differential expression. The analytical procedure is the same irrespective of whether a genome or a transcriptome was used as the reference. In both cases, the result is a table wherein each row represents a unique sequence, and each column represents a unique sample and replicate. Each cell in this table indicates the number of reads assigned to that particular sequence in that particular sample-replicate. A statistical approach is adopted wherein the mean value of the read counts for each sequence over the sample replicates is compared between the conditions of interest.

There are a number of packages in various programming languages that are capable of performing DE analysis. Although the methods they implement differ [91], they all perform the following tasks: (1) normalizing the read counts to account for differences in sequencing depths between the samples [116], (2) noise reduction [117] (optional), (3) fitting a read counts distribution to the data, and using it to test differential expression of each gene between the conditions of interest and (4) correcting the produced *P*-values for multiple testing.

Whether or not a gene or transcript has been differentially expressed is indicated through a set of numerical values, of which two are of particular importance in the context of biological interpretation. The aforementioned corrected *P*-values indicate whether the difference in expression of a gene/transcript between two conditions is *statistically significant*. A small *P*-value indicates that the probability of the read counts being different between the two conditions purely due to chance is very low: i.e. it is highly probable that the source of the difference is a biological phenomenon. The log2FoldChange value describes the *magnitude* of the difference in expression: one of the two conditions is taken as the baseline and the change in expression in the other is calculated relative to this. As the name suggests, this is the log$_2$ value of the ratio of the mean counts of the two conditions being compared. A positive log2FoldChange (lfc) value indicates upregulation, and a negative value indicates downregulation with respect to the condition being adopted as the basis for comparison. Lowly expressed genes/transcripts tend to have higher variability in their support, leading to the lfc being overestimated for these. Therefore, an important—but often overlooked—step is to correct the lfc estimates with a shrinkage algorithm (such as apeglm [118] or ashr [119]) before using them for biological interpretation. It is conventional to consider only those genes/transcripts that have a certain level of statistical significance and magnitude of difference in expression (e.g. *P*-value $<= 0.05$ and log2FoldChange  $\notin \{-1, 1\}$) as being differentially expressed.

The most popular packages for DE analysis today have all been developed for use with the R [120] statistical programming language. The three popular packages in this regard are DESeq2 [121], edgeR [122] and limma [123, 124]. Among the three packages, DESeq2 appears to be the most conservative, detecting fewer differentially expressed genes in general in comparison to edgeR and limma [125]. A number of tools have also been developed to facilitate import/export of the requisite data into the R environment, and pre-process them for DE analysis. In this regard, we recommend the use of tximport [126] which is capable of preparing data from commonly used abundance estimators such as RSEM, Kallisto and Salmon for analysis with all three aforementioned DE packages. Other packages to facilitate DE analyses exist. For example, RUVSeq [127] can be used to correct for batch effects in the data, SARTools [128] can be used to obtain standardized DE analysis templates, MetaCycle [129] can be used to perform time-series RNA-seq analysis [130] and consensusDE [131] can be used to perform DE analysis employing a multi-algorithmic approach.

Finally, we like to point out that DE analysis has been covered in much detail elsewhere (e.g. *Conesa et al.* [91], *Van den Berge et al.* [132], *Schurch et al.* [133], *Finotello and Di Camillo*[134], *Li and Li*[135], *McDermaid et al.* [124]), and we defer to those publications for an in-depth discussion of the topic. In specific, *McDermaid et al.*[124] offer an excellent overview of DE analysis packages, and *Conesa et al.* [91] offer a comprehensive review plus recommendations for RNA-seq experiments with a focus on DE applications.

**Links:**

apeglm - https://bioconductor.org/packages/release/bioc/html/apeglm.html

ashr - https://github.com/stephens999/ashr, https://cran.r-project.org/web/packages/ashr/index.html

consensusDE - https://bioconductor.org/packages/release/bioc/html/consensusDE.html

DESeq2 - https://bioconductor.org/packages/release/bioc/html/DESeq2.html

edgeR - https://bioconductor.org/packages/release/bioc/html/edgeR.html

limma - https://kasperdanielhansen.github.io/genbioconductor/html/limma.html  https://bioconductor.org/packages/release/bioc/html/limma.html

MetaCycle - https://cran.r-project.org/web/packages/MetaCycle/index.html, https://github.com/gangwug/MetaCycle

RUVSeq - https://bioconductor.org/packages/release/bioc/html/RUVSeq.html

SARTools - https://github.com/PF2-pasteur-fr/SARTools

tximport - https://github.com/mikelove/tximport

## RNA classification

The method used to isolate, enrich and sequence a sample will affect the composition of the sequencing data in terms of the types of RNA species represented and their relative abundances [12, 14, 39, 136]. Most RNA-seq studies are interested in protein-coding transcripts, and appropriately use Poly-A capture—or rRNA depletion if focusing on prokaryotes—to enrich for mRNA molecules [14]. Such enrichment is especially necessary to diminish the abundance of rRNAs, which would otherwise represent a majority of the sequenced molecules [12, 39]. However, ‘contaminant’ RNA species can still make their way into the assembled data, despite applying pre-assembly filtering measures to exclude such species (see section 2). *In silico* RNA sequence classification can therefore be used to enrich the data post-assembly for the RNA of interest.

*In silico* classification is mostly performed ad hoc. If the purpose of classification is simply to sieve out mRNAs from the rest, this can be easily achieved by assessing the coding potentials of the assembled contigs using tools like CPC2 [137] or CPAT [138], and retaining only those contigs that score above some satisfactory coding potential threshold. An alternative path to the same end result would be to retain only those contigs that produce a peptide sequence when passed through a translation tool (see Section ‘Sequence translation’ for an overview of these tools).

More granular classification can be obtained by using the tool Infernal [139]. Infernal (INFERence of RNA ALignment) is capable of classifying input sequences into rRNAs, tRNAs and lncRNAs on the basis of sequence comparison against a reference database. Infernal uses co-variance models and the Rfam [41] database to classify the input sequences. By process of elimination (i.e. whatever is not an rRNA/tRNA/lncRNA), Infernal can indirectly help identify mRNAs from the assembly. The Infernal-Rfam workflow’s output can be difficult to parse for the purpose of RNA classification, and we therefore recommend reading this portion (https://docs.rfam.org/en/latest/faq.html#rfam-and-infernal) of the Rfam documentation.

If only rRNA classification is needed, barrnap is a fast and lightweight option. It uses the Rfam, SILVA [42] and NCBI RefSeq mitochondrial [140] databases to identify and annotate rRNAs. If SortMeRNA or other an equivalent classification tool has been deployed before assembly, then the reads will have to be assembled separately prior to annotation with barrnap. An older tool, RNAmmer [141], continues to be available for use as in stand-alone and web server formats for the purpose of rRNA identification also.

lncRNAs are RNA molecules longer than 200 nucleotides with low coding potential [142, 143]. These molecules typically play regulatory roles in the cell, either directly as RNA entities, or via the short ‘micropeptides’ that result from their translation [5, 143]. Classification/identification of lncRNAs is typically achieved by elimination; that is, all sequences that are of sufficient length and have not been classified as some other RNA species (e.g. mRNA or rRNA) and have a low coding potential must be lncRNAs. We direct the interested reader to consult Motheramgari et al. [144] and Kashyap et al. [145] for demonstrations of elimination techniques for classifying lcnRNAs.

Finally, RNA classification can also be achieved via sequence searches against appropriate databases (e.g. NCBI RefSeq RNA). We discuss sequence searches in Section ‘Identity assignment via homology transfer’.

**Links:**

barrnap - https://github.com/tseemann/barrnap

CPAT - https://github.com/liguowang/cpat, http://lilab.research.bcm.edu/ (web server)

CPC2 - https://github.com/gao-lab/CPC2_standalone, http://cpc2.gao-lab.org/ (web server)

Infernal - http://eddylab.org/infernal/, https://github.com/EddyRivasLab/infernal

NCBI RefSeq - https://www.ncbi.nlm.nih.gov/refseq/

Rfam - http://rfam.xfam.org/, https://docs.rfam.org/en/latest/index.html

SILVA - https://www.arb-silva.de/

RNAmmer - http://www.cbs.dtu.dk/services/RNAmmer/ (web server, standalone download link)

## Sequence translation

A core element in the downstream analysis for RNA-seq data involves the translation of assembled sequences into their corresponding amino acid sequences, and on the nucleotide level into the protein coding sequences (CDS) not containing any untranslated regions (UTRs). A correct characterization of CDS is not only important for profiling the protein-coding fraction of a transcriptome, but also for an accurate classification of UTRs and non-coding sequences/regions which may be of interest in the context of gene regulation [146].

There are a number of tools that can predict coding regions, and subsequently translate them into amino acid sequences. These tools are based on probabilistic models that take nucleotide composition as well as length of open reading frames (ORFs) into account for their predictions [45]. Tools like TransDecoder [45], Prodigal [147], GeneMarkS-T [148] and CodAn [146] are so-called *ab initio* predictors, meaning that the prediction model is based on self-training methods. This includes identifying a certain number of long ORFs from within the assembly, which serve as test set for predicting CDS from the remaining contigs afterwards [146, 148]. A recently released tool named BOrf [149] focuses on ORF prediction for strand-specific RNA-seq, but also performs acceptably with non-specific data.

Alternatively, translations can also be obtained by simply scanning the inputs for ORFs in all six reading frames, and reporting all translations. There are many tools that can perform this including the web-based NCBI ORFfinder and EBI EMBOSS-Sixpack [150], as well as esl-translate from the HMMER suite [151] and extractorfs from MMseqs2 [108–111]. Finally, a novel approach to recovering a protein data set from RNA-seq data is presented in the tool PLASS [152] which directly scans short reads for ORFs and extends these into amino acid contigs by examining overlaps between the translations.

CDS prediction and sequence translation is not always performed, but it is recommended as sequence comparisons (necessary for annotation, see Section ‘Transcriptome functional annotation’) are more sensitive with protein sequences rather than with the corresponding nucleotide counterparts. We direct the interested reader to refer to Section 4.2, Chapter 4 of Koonin and Galperin [153] and Pearson [154] for explanations.

**Links:**

BOrf - https://github.com/betsig/borf

CodAn - https://github.com/pedronachtigall/CodAn

EMBOSS-Sixpack - https://www.ebi.ac.uk/Tools/st/emboss_sixpack/

esl-translate - http://hmmer.org/, https://github.com/EddyRivasLab/easel

GeneMarkS-T - http://exon.gatech.edu/GeneMark/license_download.cgi

ORFfinder - https://www.ncbi.nlm.nih.gov/orffinder/ (web server)

PLASS - https://github.com/soedinglab/plass

Prodigal - https://github.com/hyattpd/Prodigal

TransDecoder - https://github.com/TransDecoder/TransDecoder

## Transcriptome functional annotation

Once a transcriptome has been assembled and quality controlled, its sequences can be studied to elucidate the functionality they individually and collectively represent in the circumstances under which the data were obtained. For instance, an assembled transcript that is overrepresented in the assembled transcriptome may code for a structural protein, indicating that the cell was in a state of enhanced structural modification activity at the time of sampling.

**Functional annotation** is the process of inferring and assigning information concerning the biological functionality of the sequence using *in silico* methods. Functional annotation is usually understood to refer to the annotation of mRNAs, as it is the proteins, which these sequences are translated into, that carry out the various activities within the cell (and hence contribute to the functioning of the cell). As such it can be argued that the process of functional annotation begins with RNA classification and amino acid sequence prediction (Sections ‘RNA classification’ and ‘Sequence translation’). However, as these steps do not yield information regarding the exact functionality of the transcripts, we do not include them under the aegis of functional annotation.

**Figure 4:**
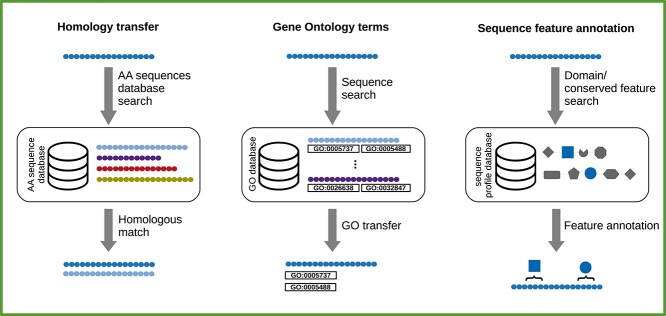
Transcriptome functional annotation comprises of techniques to assign human-comprehensible identifiers and functional characteristics to the transcripts. It includes searching for homologs based on sequence similarities and identifying assembled sequences (homology transfer), domain and other sequence feature identification (sequence feature annotation) and assigning standardized descriptors for the sequences’ biological properties (Gene Ontology terms).

There appears to be no given definition for what constitutes a standard approach to transcriptome functional annotation. A survey of relevant literature reveals that a variety of methods have been adopted in the past. For instance *Chabikwa et al.* [20] only used homology transfer (see Section ‘Identity assignment via homology transfer’), while *Sayadi et al.* [155] used a combination of different approaches to annotate their transcriptome. Based on a review of 18 papers describing annotations of *de novo* assembled transcriptomes (Table S1), we describe the transcriptome functional annotation procedure as comprising of the following steps (see also Figure 4):

- Homology transfer and identity assignment via sequence search.
- Sequence feature annotation.
- Gene ontology (GO) and biochemical pathway annotation.

We caution that these aforementioned steps are not necessarily independent nor strictly compartmentalized. For instance, sequence features *can* be annotated based on homology transfer, and need not always be performed as an independent step. Nor is it the case that all three indicated steps are mandatory to establish an annotated transcriptome. Instead, the objective is to delineate the myriad of aspects involved in transcriptome annotation—and introduce the associated tools and resources—in a succinct and concise manner.

### Identity assignment via homology transfer

Homology transfer can be considered the most basic form of transcriptome annotation. Here, a descriptive identity (e.g. ‘Protein kinase’) and functional properties are assigned to a hitherto undecorated sequence on the basis of a sequence search. In this method the assembled sequences are supplied to sequence search tools as queries. A database of well-annotated reference sequences are provided as the targets. The tools then use heuristic methods [156] to find matches between these inputs. Typically, each query has more than one matched  target. The best match is usually chosen based on the significance of the so-called e-value (more on this below). This leaves each query with a single match, whose identity and annotation are assigned to the query. It is appropriate to transfer annotations in this manner because the e-value is an indicator of the likelihood of the observed sequence similarity arising purely by chance [154]. In other words, a sufficiently low e-value (e.g. 0.00000001) is indicative of homology (shared evolutionary ancestry) which subsequently implies conserved function [154, 157]. Hence the name ‘homology transfer’ [154].

Homology transfer can be performed both with nucleotide sequences as well as (translated) protein sequences from transcriptomes. Proteins are more conserved than their corresponding mRNA sequences (see Chapter 4 of *Koonin and Galperin* [153]). Protein sequence searches are also more sensitive and faster, due to the expanded alphabet of 21 amino acids and shorter sequence length in comparison to their nucleotide counterparts. Further most functional properties (e.g. enzymatic domains) are only really meaningful in the context of a protein sequence. Because of these reasons, it is customary to either use translated searches, or pre-translated sequence sets (see Section ‘Sequence translation’), for functional annotation.

In addition to identifying homologs to the sequence, sequence features such as domains can also be transferred if the sequences are similar enough (if, for instance, they have the same length). However, such annotations would be insufficiently resolved as they would have been transferred only on the basis of sequence similarity. For instance, although two sequences are highly similar, they might not necessarily share all domains, and annotating one of them with the domains of the other on the basis of similarity alone could yield erroneous domain attributions.

**TOOLS:** The most commonly used sequence search tool for this purpose is the famous BLAST [158] suite (more precisely BLAST+ [159]). BLAST comprises of several sub-tools specialized for different types of search strategies. blastn can be used to perform searches with nucleotide sequence queries versus nucleotide sequence targets. blastp is its counterpart for amino acid queries and targets. The suite can also perform translated searches with blastx. Here, query nucleotide sequences are translated and searched against an amino acid sequence targets database. The inverse search operation (amino acid queries versus nucleotide targets) can be performed with tblastn. The suite offers rpsblast and rpsblastn to facilitate identification of conserved domains in amino acid and nucleotide queries, respectively. Finally, two options are offered by BLAST for high sensitivity searches. deltablast can perform highly sensitive searches with amino acid queries against amino acid databases. psiblast can be to identify protein homologs for amino acid queries against a database of amino acid targets using sequence-profile searches.

Although BLAST is the mainstay of sequence search tools, it is very slow, and does not scale well in terms of speed with growing input size. For instance, *Buchfink et al.* [160] indicate blastp running on ca. 21 000 CPUs would take around 2 months to scan 280 million queries against 40 million targets. Given that *de novo* transcriptomes may contain upwards of 100 000 transcripts to annotate, BLAST becomes an infeasible option—especially as a part of larger workflows. Luckily, alternatives to BLAST exist that are just as sensitive but magnitudes faster. Two such tools are discussed in the next few paragraphs below.

Diamond [160] is a special-purpose tool that is exclusively geared toward searching against protein databases. As of version v2.0.9.147, Diamond is as sensitive as blastp while being 80$\times $ faster. The tool is an almost drop-in replacement for blastp, both due to its speed, and due to the fact that it mimics the BLAST command line function calls and output formats. The main drawback of the tool is that it can only operate with amino acid sequences as targets. However, it does accept both nucleotide and protein queries. Therefore, it is a great choice for performing protein versus protein (or translated nucleotide versus protein) searches while annotating *de novo* assembled transcriptomes.

The other main alternative to BLAST is MMseqs2 [108–111] (Many-against-Many sequence searching). In some senses, it is the more equivalent alternative to BLAST as it is also a modular software suite in its own right with extensive capabilities. MMseqs2 supports nucleotide and amino acid sequences as both queries and targets, and supports translated searches via a bespoke search module. Although not nearly as fast as Diamond at equal levels of sensitivity, MMseqs2 is still 8–10$\times $ faster than BLAST at comparable levels of sensitivity. One drawback of MMseqs2 is that it uses its own database format which is incompatible with the BLAST database format. However, it can present outputs in the default BLAST format. But on the other hand MMseqs2 offers sequence–sequence search, sequence–profile search, sequence clustering and taxonomy assignment, making it a one-stop solution transcriptome annotation workflows. For instance, it can be used to replace CD-HIT for clustering and BLAST for sequence search.

**DATABASES:** The quality of annotation via homology transfer depends upon the quality of the reference databases used. It is advisable to use multiple databases encompassing different standards of curation and taxonomic scope. While a single database of references from closely related species will potentially result in fewer false annotations, a database that is taxonomy-agnostic will be invaluable in annotating novel sequences that might have otherwise been missed.

There are several general-purpose sequence databases which can be used in their entirety as reference databases, or as sources for a manually curated reference sequence set. NCBI’s [161] NR (protein) and NT (nucleotide) are non-curated, and are the largest sequence databases available today. For a well-curated set, the non-redundant NCBI  RefSeq database might be preferable. The UniProt [162] consortium’s Swiss-Prot database contains the highest quality, manually curated protein sequence set available anywhere. It can be considered the gold standard annotation source. The UniProt/TrEMBL database is the uncurated counterpart with a larger number of sequences. If all UniProt sequences are desired, the UniRef [163, 164] series of databases may be of interest, which represent subsets obtained by clustering at various levels of sequence identity. There are also taxon-specific databases maintained by various consortia. Some examples include FlyBase [165] (*Drosophila*), WormBase [166] (nematodes) and PLAZA [167, 168] (plants). Such sequence repositories are best found by reviewing relevant literature.

**Links:**

BLAST - https://blast.ncbi.nlm.nih.gov/Blast.cgi (web server), https://ftp.ncbi.nlm.nih.gov/blast/executables/blast+/LATEST/ (standalone tool download page)

Diamond - https://github.com/bbuchfink/diamond

FlyBase - https://flybase.org/

MMseqs2 - https://github.com/soedinglab/MMseqs2, https://search.mmseqs.com/search (web server)

NCBI RefSeq - https://www.ncbi.nlm.nih.gov/refseq/, https://ftp.ncbi.nlm.nih.gov/refseq/release/ (FTP)

NCBI NR and NCBI NT - https://ftp.ncbi.nlm.nih.gov/blast/db/FASTA/ (FTP)

PLAZA - https://bioinformatics.psb.ugent.be/plaza/

UniProt - https://www.uniprot.org/

WormBase - https://wormbase.org/

### Sequence feature annotation

A very important aspect of annotation is the precise identification of functional sequence features such as protein domains, disordered regions, motifs, transmembrane helices and so forth. While these can be annotated via homology transfer, the process can be error prone and have poor resolution. For instance, an assembled partial sequence may be identified as being homologous to a protein containing a bZIP domain, without explicitly aligning to the sub-sequence corresponding to that domain. Annotating the sequence with a bZIP domain would be erroneous in this case. Therefore, approaches that explicitly detect the presence of such features is preferable for the purposes of such annotations.

Sequence features such as domains are typically annotated by comparing the query sequence against databases of Hidden Markov Model (HMM) [169] representations of sequence profiles [170, 171]. Sequence profiles are compact representations of multiple sequence alignments (MSAs) [172] of protein families wherein the aligned residues correspond to domains or other conserved features. Domains on the query sequence(s) can be detected by performing a sequence-profile alignment against the HMMs using a tool such as HMMER3 [151]. But not all sequence features are predicted this way. For instance, signal peptides are predicted by the tool SignalP using a deep learning method [173], the tool fLPS [174] uses a statistical approach called probability minimization to predicted biased regions in amino acid sequences and protein motifs [175] can be predicted using simple pattern matching techniques. As such a large variety of tools and databases exist to facilitate annotation of various sequence features.

InterProScan [176] is a metatool that integrates a number of feature prediction methods, databases and analyses into a single user-friendly interface. It negates the need for having to install and maintain a variety of databases and tooling manually. In addition to annotating protein functional and structural domains, it can also be used to classify sequences (e.g. into protein families on the basis of gene ontology), and detect transmembrane and disordered regions. InterProScan can be run on both nucleotide and amino acid sequneces. A web server version of the tool is also available. The interested reader can refer to https://interproscan-docs.readthedocs.io/en/latest/HowToRun.html#included-analyses for a complete list of analyses included in the tool.

A large number of resources are available for annotating a myriad variety of sequence features. It is advisable to scan recent literature for relevant tools for niche use-cases. In addition to InterProScan, we would like to highlight two tool repositories that should be of interest. The software portal at DTU Health Tech (https://services.healthtech.dtu.dk/software.php) hosts a number of useful annotation tools including predictors for post-translational modifications. The European Bioinformatics Institute (EMBL-EBI) provides a wide variety of tools and data resources at https://www.ebi.ac.uk/services that may also be of interest in the context of sequence annotation.

The huge variety of annotatable sequence features can be overwhelming to choose from. It is advisable to only annotate those features that will be of interest for downstream applications. For a standard transcriptome annotation workflow, it should suffice to annotate protein functional domains (e.g. against PFam [177]) and structural domains (e.g. against CATH-Gene3D [178, 179]) using a tool such as InterProScan.

**Links:**

CATH-Gene3D - https://www.cathdb.info/

fLPS - https://biology.mcgill.ca/faculty/harrison/flps.html, https://github.com/pmharrison/flps

HMMER3 - http://hmmer.org/, https://www.ebi.ac.uk/Tools/hmmer/ (web server)

InterProScan - https://github.com/ebi-pf-team/interproscan, https://www.ebi.ac.uk/interpro/ (web server)

Pfam - http://pfam.xfam.org/

Tools at DTU Health Tech - https://services.healthtech.dtu.dk/software.php

Tools at EMBL-EBI - https://www.ebi.ac.uk/services

### Gene ontology and pathway annotation

It is useful to assign descriptors from a controlled vocabulary (ontology) that associates the sequences with specific biological phenomena in a consistent manner. For this purpose the assembled sequences can be annotated with Gene Ontology (GO) terms [180, 181] (see *Dessimoz and Škunca* [182] for details on GO terms and their usage). There are several possibilities for annotating sequences with GO terms.

Firstly, GO terms can be transferred from homologous sequences via sequence search (Section ‘Identity assignment via homology transfer’). If InterProScan [176] (Section ‘Sequence feature annotation’) is being used, it can be asked to annotate GO terms using the --goterms commandline switch. If a standalone GO annotation tool is required, the functional annotation tool eggNOG-mapper [183, 184] is one of the only open source, free-to-use options (see Section ‘Transcriptome annotation suites’). Alternatively, the Orthologous Matrix Browser (OMA Browser) [185] offers some web-based options for GO annotation. For those willing to pay a licensing fee (or use a free version with limited capabilities), the BLAST2GO [186] functional annotation suite is available as an alternative.

Pathway annotation refers to assigning the sequences to one or more biochemical pathways. There are two popular pathway annotation databases: the Kyoto Encyclopedia of Genes and Genomes (KEGG) [187–189] and reactome [190]. The latter is more human-centric, making KEGG the more widely used database, especially for non-model organisms. InterProScan, eggNOG-mapper, and BLAST2GO all transfer pathway annotations alongside GO annotations, so no additional tooling is usually necessary. The annotation suite Trinotate mentioned in Section ‘Transcriptome annotation suites’ also provides GO annotations by transitive assignment from Swiss-Prot to BLAST hits. If only a homology transfer (Section ‘Identity assignment via homology transfer’) is being performed, pathway annotations can be transferred akin to GO annotations. Pathway assignments can also be obtained independently by annotating the transcriptome via the KEGG Automatic Annotation Server (KAAS) or reactome web servers, respectively. For KEGG annotations, the GhostKOALA [191], BlastKOALA [191] and KofamKOALA provide additional functional annotation options.

**Links:**

BLAST2GO - https://www.blast2go.com/

eggNOG-mapper - https://github.com/eggnogdb/eggnog-mapper, http://eggnog-mapper.embl.de/ (web server), http://eggnog5.embl.de/#/app/home (eggNOG database)

InterProScan - https://github.com/ebi-pf-team/interproscan, https://www.ebi.ac.uk/interpro/ (web server)

KAAS - https://www.genome.jp/kegg/kaas/

KEGG - https://www.genome.jp/kegg/

BlastKOALA - https://www.kegg.jp/blastkoala/

GhostKOALA - https://www.kegg.jp/ghostkoala/

KofamKOALA - https://www.genome.jp/tools/kofamkoala/

OMA Browser - https://omabrowser.org/oma/home/

reactome - https://reactome.org/ (including analysis web server)

## Transcriptome annotation suites

Transcriptome annotation involves a number of different tools and databases, dealing with which can quickly become a cumbersome task in of itself. In recent years, a number of annotation *suites* have been developed with the objective of making this an easier process. In most cases, these wrap existing tools into a single easy-to-use interface while adding features useful for transcriptome annotation (e.g. expression-based filtering). In all cases, some combination of sequence identity assignment, sequence feature detection and gene ontology/pathway assignment are performed as a part of the annotation procedure (as described in Section ‘Transcriptome functional annotation’).

Trinotate [192] is arguably the most well-known open source, free-to-use annotation suite. It accepts both nucleotide and amino acid sequences as inputs. The former are translated using TransDecoder. It uses BLAST+ for homology search, and HMMER3 (against Pfam) for sequence feature annotation. Optionally, it can run rnammer for RNA classification, Signalp for signal peptide identification and tmhmm [193] for predicting transmembrane domains. User-supplied databases are also accepted in addition to the default UniProt/Swiss-Prot database for the homology search step. GO annotations are provided via transitive assignments from top homology search hits. Trinotate uses a SQLite to collate and summarize the results. A graphical user interface (GUI)–TrinotateWeb–is available for visualizing and navigating the results. The tool’s main advantage is its tight integration with the Trinity assembler.

Dammit is a popular alternative to Trinotate. The tool is written in python, and is available via the conda package management system. This ensures ease of installation (all dependencies come pre-packaged), upgrade and use. The tool offers a default set of reference databases (including NCBI NR) but also accepts user-supplied ones. Rather than relying on homologs for annotation, Dammit searches with a specialized reciprocal best hit method for orthologs (using LAST), while accounting for issues caused by the presence of transcript isoforms in the assembly. Annotating via orthology is superior as these are genes related by speciation that have the same function as opposed to generic homologs which may be paralogs where function need not be conserved (see *Altenhoff et al.* [194–196]). Sequence feature annotation is performed using HMMER3 against the Pfam database, and RNA classification using Infernal against Rfam. BUSCO scores are included in the annotation report as the tool is a part of the pipeline as well.

EnTAP [197] is a recently released annotation suite with a focus on *de novo* assemblies of non-model eukaryotes. It uses the fast Diamond aligner internally for identity assignment via homology, and uses eggNOG-mapper for gene ontology annotation. Sequence features can be annotated via homology transfer or via an optional InterProScan run. EnTAP accepts both nucleotide and amino acid sequences. With the former it is possible to filter on the basis of expression level using an FPKM threshold before translation with TransDecoder or GeneMarkS-T. No nucleotide-level annotations (e.g. RNA classification) are possible as of the current release (v0.10.8). EnTAP offers several unique features helpful for annotations of non-model organisms. For instance, if NCBI or UniProt are used, annotations can be enriched and/or filtered out on the basis of taxonomic scope with regards to the species being annotated. e.g. metazoan matches in the sequence search can be prioritized while bacterial sequences can be indicated as contaminants when annotating an arthropod. The tool also presents the annotation results in multiple formats, and incorporates useful statistics and figures.

Annocript [198] is an annotation suite built around BLAST+. Annotations via homology transfer are based on either user-defined reference sets or a default UniProt database. Sequence features are annotated using rps-blast and NCBI’s Conserved Domain  Database (CDD) [199]. Annotations are stored and summarized via a MySQL database. Annotations include GO terms and pathways.

Sma3s (Sequence massive annotator using 3 modules) [200] is a general purpose annotation suite that can also be used with transcriptomes. Written in perl, its only dependency is the BLAST+ suite. Annotations are made against the UniProt/UniRef90 database using homology transfer, and include sequence identifiers, GO terms, as well as pathways. The tool can also be used with custom reference databases.

TOA (Taxonomy-oriented Annotation) [201] and TRAPID 2.0 [202, 203] are transcriptome annotation platforms with a focus on plant species. The former is a platform-agnostic, offline tool while the latter is a web server that requires registration. Both tools use methods similar to the more mainstream annotation suites, but restrict the reference databases to select plant-related ones.

The Transcriptome Computational Workbench  (TCW) [204] is an interesting annotation tool written in Java that can not only annotate multiple transcriptomes but can also perform comparisons between them. In addition, the tool has built-in functionality to carry out differential expression analysis. TCW is arguably one of the oldest transcriptome annotation tool in existence, having undergone continuous development since 2013 [205].

Given the increasing complexity of RNA-seq experiments and concerns regarding reproducibility, the use of bioinformatics workflow managers (see Section ‘Workflow managers’) to orchestrate reproducible and extensible workflows has become a popular approach. The domain of *de novo* transcriptome assembly and annotation has not been exempted from this revolution. FA-nf [206] and transXpress are two such annotation platform. The former is functional annotation suite billed as being specialized for non-model organisms, while the latter is a complete assembly and annotation pipeline that can be operated almost turnkey.

The functional annotator eggNOG-mapper deserves a honorable mention here, since it provides a full set of relevant annotations including orthologs, domains (from Pfam), GO terms and pathways despite not being billed as a transcriptome annotation tool. Likewise, the Orthologous Matrix (OMA) Browser mentioned in Section ‘Transcriptome annotation suites’ offers a stand-alone option (OMA StandAlone [207]). This tool can perform orthology predictions and GO annotations, but does not provide domain annotations. Other general purpose functional annotation tools such as the WebMGA [208] web server and PANNZER2 [209] can also be used to annotate transcriptomes via their translated sequence sets.

Finally, BLAST2GO is perhaps the most popular transcriptome annotation tool. It is not open-source and requires a paid subscription for full functionality. The tool is built around annotation transfer based on BLAST+ homology searches, coupled with a user-friendly GUI. A ‘basic’ version with limited capabilities is available for free use. The BLAST2GO tool is a part of the larger OmicsBox (https://www.biobam.com/) bioinformatics platform which offers a wide variety of bioinformatics-related tools and analysis (including *de novo* transcriptome assembly).

**Links:**

Annocript - https://github.com/frankMusacchia/Annocript

Dammit - https://github.com/dib-lab/dammit, http://dib-lab.github.io/dammit

eggnog-mapper - https://github.com/eggnogdb/eggnog-mapper, http://eggnog-mapper.embl.de/ (web server)

EnTAP - https://github.com/harta55/EnTAP

FA-nf - https://github.com/guigolab/FA-nf/tree/0.3.1

OMA StandAlone - https://omabrowser.org/standalone/

PANNZER2 - http://ekhidna2.biocenter.helsinki.fi/sanspanz/

Sma3s - https://github.com/UPOBioinfo/sma3s, http://www.bioinfocabd.upo.es/web_bioinfo/sma3s

TCW - http://www.agcol.arizona.edu/software/tcw/, https://github.com/csoderlund/TCW

TOA - https://github.com/GGFHF/TOA

TRAPID 2.0 - http://bioinformatics.psb.ugent.be/trapid_02/

transXpress - https://github.com/transXpress/transXpress-nextflow (**Nextflow** version), https://github.com/transXpress/transXpress-snakemake (**Snakeake** version)

Trinotate - https://github.com/Trinotate

WebMGA - http://weizhong-lab.ucsd.edu/webMGA/server/

## Comparing transcriptome assemblies

A set of assemblies can be used in a comparative transcriptomics approach, for instance, to identify conserved genes or specific gene expression patterns associated with different organisms of interest. If more than two organisms are studied, a first step in such analysis consists in constructing a phylogenetic tree describing the evolutionary relationship between the representative transcriptomes. To do so, a suitable approach taking advantage of the previously identified BUSCO genes (during post-assembly quality control, see Section Post-assembly quality control) can be used [77]. BUSCO genes have been curated to represent a conserved set of slowly evolving housekeeping genes which can be used for phylogenetic analysis. A common approach consists of retrieving the translated transcript sequences associated with each BUSCO gene in the different transcriptomes. Then, MSAs are performed with tools like MAFFT [210] or FAMSA [211], for each house-keeping gene with a single copy in every transcriptome of interest. These MSAs can then be used to construct phylogenetic trees. There are many tools that can be used to build such trees, e.g. RAxML [212]. A representative consensus species tree reflecting the phylogeny of the total set of single copy BUSCO gene trees can then be reconstructed, using dedicated methods like ASTRAL-III [213]. To analyze the presence or absence of genes across multiple transcriptomes, and be able to compare the expression of the conserved ones, it is essential to identify orthologs and paralogs within the studied data set [194]. While numerous orthology prediction methods have been developed over the last two decades, OrthoFinder [214] has become widely adopted and quasi-standardized, due to its speed and ease of use. The tool uses a combination of sequence clustering and tree building methods to group sequences (from all input samples) into orthogroups. Orthogroups basically represent collections of sequences that are related at *their* root node by speciation [194]. From these data, OrthoFinder is able to estimate gene copy numbers, orthogroup trees, a consensus species tree and other useful evolutionary data (e.g. sets of single-copy orthologs, pairwise orthologs, etc.). A recent alternative to OrthoFinder is the very fast JustOrthologs method [215]. As it performs comparisons between pairs of organisms, it is especially adapted to the study of pairs of transcriptomes, but its use can be extended to the comparison of numerous ones using the associated CombineOrthoGroups script, which combines pairs of orthologs into orthogroups. Likewise, the OMA StandAlone [207] function annotation tool can also perform comparisons between the input assemblies.

While BUSCO-derived phylogenies and orthlogy prediction have been commonly adopted in the last decade for comparing assembled transcriptomes, a recent study addressed the biases and limits of such approach [216]. By comparing low- and high-quality transcriptome assemblies (scored with TransRate [80], see Section ‘Post-assembly quality control’), it highlighted that some important skews in phylogenetic and orthology prediction data can come from using low-quality assemblies. Not to draw any wrong biological interpretation from comparative transcriptomics, it is therefore important to consider assembly quality at every point in such an analysis.

**Links:**

BUSCO - https://busco.ezlab.org/

FAMSA - http://sun.aei.polsl.pl/REFRESH/famsa

JustOrthologs - https://github.com/ridgelab/JustOrthologs

MAFFT - https://mafft.cbrc.jp/alignment/server

OMA StandAlone - https://omabrowser.org/standalone/

OrthoFinder - https://github.com/davidemms/OrthoFinder

RAxML - https://raxml-ng.vital-it.ch

## Workflow managers

Modern biological science is high-throughput and highly data-driven. Investigations often deploy composite computational analyses using multiple tools to process the data. A collection of such tools/programs organized in a specific manner to produce results from which biological inferences can be drawn is known as a workflow or pipeline [217]. Similar to how a ‘wet-lab’ protocol represents the set of steps required to transform a ‘raw’ sample into comprehensible output (e.g. sequencing an RNA molecule), a bioinformatics workflow/pipeline represents an equivalent collection of steps to do the same with digital data [218] (e.g. identifying an RNA sequence as an mRNA). A workflow consisting of a small number of tools and/or a small amount of data can be handled by the investigator(s) by executing each step/tool manually. However, for projects dealing with large volumes of data and/or a complex, interconnected collection of tools, automatization of the workflow becomes unavoidable [219].

It is in such cases that workflow managers/workflow management systems (WfMS) become useful. A WfMS is a specially designed programmatic framework that can be used to automate a pipeline consisting of numerous steps that must be manually executed [217]. It allows the user to define the computational pipeline as graph wherein each node represents a particular processing step. The edges connecting the nodes are directed and represent data flowing from one node after being processed by it (its output) to another node as its input. Thus, the graph also describes the order in which the components of the pipeline will be executed. The user must define the individual steps of the workflow in terms of the inputs, expected outputs and the tool(s) required to generate them. Typically, it is required that the user specifies the exact command to run the tool using placeholder values to define the inputs and outputs (for example mytool inputfile outputfile). The workflow manager then handles the execution of the pipeline. This includes allocating resources (processing threads, memory, etc.) and deducing the order in which the individual commands have to be executed. The advantage of using a workflow manager is that analyses become optimized, especially when dealing with large volumes of data and metadata as the execution details are abstracted away from the user [217]. Further, as the user only needs to define the workflow but not the specifics of execution, the same pipeline can be executed on a local server, cluster or in the cloud, making pipelines scalable and easy to prototype [220]. Finally, using a workflow manager also makes analyses reproducible, shareable and easy to run as workflows can be run anywhere, and can often also install the correct versions of the tools by themselves [221]. This also makes bioinformatics accessible—as non-experts can avail themselves of pre-existing workflows for their own research [222].

Workflow managers can be sorted into two groups—command-line interface-based (CLI) and GUI-based. The two groups primarily differ in how the workflow manager itself is presented to the user. A CLI-based WfMS is a command-line program that executes a text document (script) describing the analytical workflow. Most WfMS have a particular programming language they can recognize, and the script must be written in this language. In comparison, a GUI-based manager exposes the same equipment to the user via a point-and-click environment. Users are able to construct workflows by dragging and dropping and interconnecting icons representing tools and data. Experienced users will save time by working with CLI managers, since writing a command for a particular process is faster than manually navigating the interface panels of a GUI program. On the other hand, GUI WfMS are much more user-friendly and do not demand knowledge of programming.

Recent publications [217, 222, 223] indicate that the most popular WfMS today include Nextflow [224], Snakmake [221] and Common Workflow Language (CWL) [225]. All three are CLI-based, open-source and free-to-use, but have their differences.

Snakemake is based on Python which is among the most popular programming languages [226]. As a result of Python’s user-friendly syntax, workflows written in Snakemake are not only very readable but also approachable for beginners. In addition to facilitating custom workflows, users can also import external pipelines, and merge and edit them depending on their needs [221]. In this regard, some so-called wrapper scripts are offered through the Snakemake Wrapper Repository (https://snakemake-wrappers.readthedocs.io/en/stable/) that are templated for common bioinformatics tasks. Snakemake pipelines are portable and scalable. They can be executed on a wide range of environments starting from single-core workstations to HPC clusters [227]. The only requirements are Python and Snakemake itself.

Nextflow is a powerful WfMS based on the Groovy programming language. The central idea is that most bioinformatics tools are Unix-based, and data are passed between the tools (and processed additionally) using custom scripts often written in different languages (e.g. Python and R). Consequently, Nextflow permits chaining together scripts (and tools) written in different languages as long as they can be executed on a Unix-like operating system [228]. Like Snakemake  Nextflow is also scalable and platform-agnostic with regards to execution capabilities. A salient feature of Nextflow is nf-core (https://nf-co.re/) [229]. This is a curated repository of bioinformatics pipelines written in Nextflow that cover a range of use cases including RNA-seq, sequence assembly, phylogenetics and sequence annotation.

Common Workflow Language (CWL) is another CLI-based WfMS. However, while the previous two are focused on pipeline development, CWL also represents a set of standards defining what a workflow language should look like and contain. This is because the advent of ‘big data’ in biology has led to the introduction many WfMS implementations (not discussed here) all of which use different approaches for describing their pipelines [230]. Adherence to CWL standards would allow pipelines to be shared, easing the process of testing and comparing new methods acquired from other researchers, despite having been implemented in different WfMS [218]. CWL itself represents a set of standards, and cannot be used to draft a workflow. A set of CWL-compliant WfMS implementations—e.g. CWL-Airflow (https://github.com/Barski-lab/cwl-airflow) [230]—can be found on its website (https://www.commonwl.org/#Implementations); a reference implementation (cwltool) developed by the CWL team is also available.

Workflow Description Language [231] (WDL) is a WfMS with straightforward syntax. Analogous to CWL, it also represents a language definition and is not executable in of itself: a WDL-compliant execution engine is required to execute workflows. The two main execution engines are Cromwell and miniwdl. WDL is under active development, supports multiple programming languages and has a growing ecosystem of tools and pre-defined workflows. Finally, as suggested above, tooling to design and execute workflows (bioinformatics or otherwise) exists elsewhere—often as language-specific implementations. For instance, the Targets [232] package enables this in the R programming language popular among biologists and bioinformaticians. Such implementations permit users to design and execute workflows using a language familiar to them.

Galaxy [23] is arguably the most used web-based data analysis platform for biology [233]. It is intended to serve researchers from a broad variety of backgrounds looking to investigate large quantities of data with complex tools, even those with limited programming experience [234]. It is an open-source WfMS with a GUI that allows for work to be carried out entirely in a web browser. Galaxy is analysis-agnostic: although originally written for genomic analyses in mind, it has since been used for a vast variety of research (e.g. biophysics [235]). A large, open repository of tools contributed by the user community is available through the Galaxy ToolShed [236]; installation is easy as Galaxy automatically activates the required dependencies also. A large collection of pre-scripted workflows for a variety of common analytical tasks are also available, reducing the need for recreating boilerplate routines. The Galaxy approach also ensure easy documentation of workflows as workflow components can be directly annotated through the GUI. Needless to say, the platform ensure easy reproducibility of workflows. In addition, the platform not only takes care of resource allocation for workflow execution, but also provides the resources themselves in the event that the user is operating on the free public server (https://usegalaxy.org/). Alternatively, the platform itself is available as an open-source tool that can be downloaded, installed and configured for local use (e.g. on a personal computer or an HPC environment). A plethora of customizations to make Galaxy even more user-friendly (e.g. Galaksio [237]) are available and continue to be developed.

Although Galaxy dominates the GUI-based WfMS space, there are a few other alternatives worth mentioning. The Unipro UGENE [238] bioinformatics suite offers an integrated WfMS for constructing workflows with in-built tools. Although the suite is open source and cross-platform, it cannot be used on HPC environments. GenePattern [239] is a more equivalent competitor to Galaxy offering many of the same features including a public server and a version for stand-alone installation.

**Links:**

Cromwell - https://github.com/broadinstitute/cromwell

CWL - https://www.commonwl.org/

Galaxy - https://galaxyproject.org/ (homepage of the project), https://usegalaxy.org/ (free to use public server)

GenePattern - https://www.genepattern.org/#, https://genepattern.org/ (public server), https://github.com/genepattern (GitHub repository)

miniwdl - https://github.com/chanzuckerberg/miniwdl

Nextflow - https://www.nextflow.io/

Snakemake - https://snakemake.github.io/

Unipro UGENE - https://ugene.net/

WDL - https://github.com/openwdl/wdl

## Computational and programmatic considerations

All the necessary tools must be acquired and installed before embarking on the task of assembling and annotating a transcriptomic data set. There is a large variety of tools, all with varying levels of availability and support. It is often the case that the tool is available only on a specific operating system (OS) or requires specialized domain knowledge for installation. Executing tools can be all the more challenging than acquiring them, especially when multiple tools need to be used in concert on extremely large data sets. Assessing the computational resources for deploying these tools can also be very difficult. All these aspects invoke additional considerations that the researcher must take into account before and during the analysis. Addressing all these topics in a thorough manner is a non-trivial endeavor. However, in the interest of signposting useful resources that could be consulted, we address these in an introductory manner below.

### Operating systems, programming languages and computational resources

Most tools and software for bioinformatics and analysis in biology have been written for Unix-like operating systems (https://en.wikipedia.org/wiki/Unix-like), and are often designed to be run from within a command line shell [240]. Thus, it is preferable to have access to a computer or computing environment equipped with such an OS.

The most popular Unix-like OSes in use today are Apple’s closed-source macOS(https://www.apple.com/macos) and the various ‘flavors’ of the open-source and free-to-use Linux [241] family. Users of Microsoft Windows (https://www.microsoft.com/en-us/windows/) can install the Windows Subsystem for Linux application to avail themselves of a Unix-like environment on this operating system. macOS users have access to an in-built command line shell.

As a general recommendation, we suggest using the Linux-based  Ubuntu operating system and the included GNU Bash shell. This combination is well documented due to a large install base, and is open source, free to use and continually maintained by the developers and community. Many tools of interest are also readily available for this platform via Ubuntu’s package manager (https://ubuntu.com/server/docs/package-management), as pre-compiled binaries/executables from the developers, or as source code that can be compiled easily. External package management systems (refer Section ‘Tool management’) are also easily available for this platform.

Interfacing with one or more programming languages is an aspect potential users of RNA-seq tools will have to consider. Users will often encounter situations where the output from one tool must be fed to another tool as its input, but the output and input formats are incompatible (e.g. a table with four columns is required as an input, but it exists as a table with five columns). In such cases, the user will have to intervene and transform/manipulate the data in order to pass it on through subsequent steps of the analysis. There may also be situations where some portion of the analysis *must* be done in a programming language; for example, almost all popular DE analysis tools (see Section ‘Differential expression analysis’) are packages that must be accessed through a programming language. In any case, in the interest of reproducibility, efficiency and making problems tractable, it is advisable to become familiar with one or more programming languages.

There are a number of such languages that are popular in bioinformatics (and in biology in general). This includes the eponymous scripting language of the GNU bash shell itself, Python [242] and R [120]. Each of these have their own strengths and weaknesses. Bash is ubiquitous and powerful but has a cumbersome syntax and is only really convenient for short programs. Python is a general purpose language with a very friendly syntax, and is nearly as ubiquitous as Bash. However, its ecosystem for bioinformatics analyses is relatively limited. R is not as prevalent as the other two but is excellent for manipulating and analyzing large amounts of data. Furthermore, it is the language of choice for bioinformatics analysis due to the large number of packages and tools it supports in this regard—especially for ‘-omics’ analyses through the Bioconductor [243] ecosystem.

It is very common to see bioinformatics workflows interspersed with scripts written by the researcher. In larger analytical workflows, e.g. hundreds of samples, 20–30 different tools, bioinformatics workflow managers come into play to ensure that the procedures can be orchestrated automatically in a fully reproducible manner (see Section ‘Workflow managers’ for a brief-but-thorough introduction to this topic).

The question of computational resources is another issue that researchers must tackle in order to be effective in their analyses. Computational resources is a catch-all phrase, and has multiple aspects to it, importantly, the number of central processing units (CPUs) and their clock speeds, the amount of random-access memory (RAM) available per CPU and storage type and capacity (hard disk drives/HDDs and/or solid state disks/SSDs). A personal computer (e.g. laptop) with a dual core CPU, 8GB (Gigabytes) of RAM and a 250GB SSD is sufficient for executing a small analysis in R or python. However, it will be grossly insufficient for running a *de novo* transcriptome assembler; for instance, the Trinity assembler (see Section *De novo* transcriptome assembly) can consume upwards of 27GB of RAM during its execution [58]. The runtime—the duration it takes for the tool to finish executing—would also be excessively long with constrained resources; case in point, even with 48 CPU cores and 512GB of RAM, Trinity can take about 6–7 h (or even days) to run [58]. Likewise, storage capacity on the order of at least 1–2TB would be required. Our personal experience indicates tools such as Trinity can routinely consume several 100GBs of both disk space and RAM during execution, and produce output directories that are themselves at least 10–20GB in size. Therefore, researchers must factor in having to acquire computational resources on this order of magnitude for workflows incorporating *de novo* assemblies. Broadly speaking, there are two ways in which such resources can be requisitioned. Depending on the usage frequency, a workstation/server with the necessary capacity may be rented or purchased outright for institutional/departmental use [244]. Very often, research and educational institutions will have their own centralized computational infrastructure (e.g. high performance compute clusters) from which such resources can be requested [244]. Computational resources may also be acquired from national-scale compute infrastructure projects [245, 246], non-profit foundations that offer bioinformatic-as-a-service (e.g. Galaxy [23]; see Section ‘Workflow managers’) or private cloud compute providers (e.g. Google Cloud Life Sciences, Amazon Web Services and Microsoft Azure).

**Links:**

Amazon Web Services - https://aws.amazon.com/health/

Bash - https://www.gnu.org/software/bash/

Bioconductor - http://bioconductor.org/

Google Cloud Life Sciences - https://cloud.google.com/life-sciences

Microsoft Azure - https://azure.microsoft.com/en-us/solutions/high-performance-computing/health-and-life-sciences/

Linux - https://www.linux.org/

Python - https://www.python.org/

R - https://www.r-project.org/

Ubuntu - https://ubuntu.com/

Windows Subsystem for Linux - https://docs.microsoft.com/en-us/windows/wsl/about

### Tool management

Almost all tools indicated in this publication are available online for download and installation. In a vast majority of the cases, the tools are available via a GitHub or GitLab repository. In some instances, tools may either be found on the author’s (e.g. a particular research group) website or on other code repositories such as SourceForge. Most tools are accompanied by a descriptive academic publication that also normally indicates where the tool can be found: e.g. the publication for the rnaSPAdes assembler [56] cites https://github.com/ablab/spades. The repositories of most tools are also usually easily found via appropriate search engine queries.

Tool installation may be as simple as de-compressing and extracting from an archive (e.g. a .tar.gz file), or can be a complicated procedure that requires compilation (ref. https://www.linuxjournal.com/article/216 for an introduction to compilation). Typically both the source code for compilation as well as pre-compiled binaries targeting a few chosen platforms are made available for download by the tool developers.

However, the best method for installing tools today would be via the open-source package manager Conda. Almost all major standalone bioinformatics tools are available via the Bioconda [243] channel, and installation in most cases is as simple as creating a new conda environment and issuing the command conda install -c bioconda exampletoolname. Most dependencies (i.e. other tools/software required for operation) are also available via conda and should be installed automatically alongside. The conda package manager also permits easy updating of installed tools and packages. This is in sharp contrast to a compiled installation where an update would typically require compiling the newly downloaded source code again and also ensuring that all dependencies are also updated without compromising the functionality of the OS.

Some tools are also available as Docker and/or Singularity containers. A container is basically the software and everything that is needed to run it enclosed into a single unit (see https://www.docker.com/resources/what-container for an explanation). Bioinformatics tools made available as containers are typically those that are either too big to be shipped stand-alone, are too complicated to be installed directly by the user, or a combination of both. These are mostly tools that have a multitude of dependencies (i.e. other software) required to run and/or come with large amounts of bundled data. Docker containers require root privileges (https://www.ssh.com/academy/iam/user/root) to run while their Singularity counterparts normally do not. Root access is typically a no-go in high performance computing (HPC) environments [247], and therefore Singularity containers are more popular in that particular context. We direct readers to documentation from Docker and Singularity for instructions on how to execute containerized software.

Executing a command line tool requires an understanding of the inputs, options and outputs as related to the tool. The first point of contact for help information/documentation is typically the tool itself. Issuing the command toolname -h, toolname -help or toolname --help should print the in-built help page. Documentation can also be found in the included README files and often in the ‘wiki’ sections of the tool repositories. If tools have associated publications, these are also a good source of information and documentation. For more advanced support it may be necessary to either contact the developers/maintainers directly via e-mail or by opening an issue on the tool repository’s issue tracker. It is inevitable that the researcher will encounter non-specific (but nevertheless important) questions/issues over the course of a bioinformatics analysis. Luckily, there are several popular online communities where such topics could be raised (e.g. Biostars).

More general questions can also be addressed to members of the bioinformatics community at large via online forums like Biostars, Bioinformatics StackExchange, Biology StackExchange and StackOverflow among others.

**Links:**

Bioconda - https://bioconda.github.io/

Biostars - https://www.biostars.org/

Conda - https://docs.conda.io/en/latest/

Docker - https://www.docker.com/

GitHub - https://github.com/

GitLab - https://gitlab.com/

Singularity - https://sylabs.io/singularity/

SourceForge - https://sourceforge.net/

## What to annotate and where to publish

**What to annotate:** Sequence annotations should ultimately serve the purposes of the study. Identity assignment via homology could be considered the bare minimum, as it allows the assembled sequences to be tied to human-comprehensible identifiers. GO terms are normally annotated because these can be aggregated to reveal the distribution of the transcriptomic output over various biological aspects (e.g. what percent of the transcriptome is involved in a biological process, etc.). They are also useful for differential expression studies wherein the GO terms of differentially expressed transcripts can be aggregated to obtain an overview of which biological phenomena are being influenced (GO enrichment analysis). It is also useful to annotate functional domains against a standard database such as Pfam.

Other annotations can be performed as the need arises. For instance, the objective of the study may be to profile simple sequence repeats in the mRNA alongside establishing a *de novo* transcriptome. In this case, the assembled sequences may be passed through an appropriate tool (e.g. the MISA web server [248]) to obtain the necessary annotations in addition to the aforementioned ‘standard’ annotations. In general, once the assembled sequences have been translated, a relatively broad variety of tools become available, opening up additional avenues for sequence annotation that can be pursued as necessary. The necessary tools are best found by consulting the literature. Continuing with the example above, MISA can be found cited in a relevant study such as Pinosio et al. [249].

**Where to publish:** Typically, an assembly and annotation workflow would result in at least one FASTA file containing the assembled sequences, and at least one tabular file (e.g. a TSV file) containing one row per sequence with individual columns representing the various annotations. Almost all studies submit their raw sequencing data (i.e. the FASTQ files) and the assembly to NCBI’s Sequence Read Archive (SRA)[250], and Transcriptome Shotgun Assembly Sequence Database (TSA), respectively. In contrast annotation files do not seem to have a standard destination. Some studies prefer to upload data to research data dissemination portals such as figshare and Zenodo [251] that can generate stable Digital Object Identifiers (DOIs) [252] to the data themselves. Annotations can also be submitted to the TSA (see https://www.ncbi.nlm.nih.gov/genbank/tsaguide/), but this is allegedly a cumbersome and tedious process. In some instances, annotation files have been provided alongside the publication as a supplementary file (e.g. *Thunders, Cavanagh and Li* [253]). Depending on the volume of data, creative solutions such as hosting the annotations on the cloud may also be valid solutions.

**Links:**

Digital Object Identifiers - https://www.doi.org/

figshare - https://figshare.com/

NCBI Sequence Read Archive - https://www.ncbi.nlm.nih.gov/sra

NCBI Transcriptome Shotgun Assembly Sequence Database - https://www.ncbi.nlm.nih.gov/genbank/tsa/

Zenodo - https://zenodo.org/

## Conclusions

As *Stark, Grzelak and Hadfield* [7] highlight in their review ‘RNA sequencing: the teenage years’, RNA-seq has become a ubiquitous tool in biology, and has steadily proliferated into allied fields of research such as ecology [17]. The advent of long-read RNA-seq [254–257] has proffered exciting prospects such as direct sequencing of RNA molecules sans cDNA synthesis [258] and sequencing RNA from single cells [259]. Despite these challenges, bulk RNA-seq via short-read sequencing remains a prominent method. One reason is the sustained (and growing) popularity of *de novo* transcriptome assembly and annotation for the purposes of studying non-model organisms. Here, well-annotated *de novo* assembled transcriptomes represent an inexpensive route for thoroughly cataloging transcripts, and identifying interesting gene products.

This enduring and widespread interest has ensured an unabated deluge of ever-improving tools, databases and workflows to facilitate assembly, annotation and associated analyses. While these utilities have greatly eased the effort of scientific discovery, the staggering variety of resources available has nevertheless made the task of choosing a suitable approach for a specific research question a complex and confusing exercise. This is perhaps especially true for non-expert practitioners who now have the means to perform RNA-seq experiments entirely in-house. For these individuals, *de novo* transcriptomics holds great promise as they can now study nearly any organism(s) of their choosing.

To this end, we presented a comprehensive and beginner-friendly overview of the major processes and tools involved in *de novo* transcriptome assembly and annotation of short-read bulk RNA-seq data. We hope that this material will aid both incoming and established researchers alike in their quest to obtain high-quality transcriptomes.

**Key Points**

- *De novo* transcriptome assembly and annotation ideal for studying non-model organisms and establishing gene catalogs thereof.
- In-housing marred by overabundance of tools, paucity of authoritative literature and non-standardized workflows.
- We present a comprehensive-but-beginner-friendly step-by-step review featuring accessible conceptual explanations and an overview of popular tools.

## Supplementary Material

table_s1_bbab563

table_s2_bbab563

## Notes

The authors are comprised of students at various stages of academia with an interest in various aspects of applied bioinformatics.

**Fantin Mesny** is a PhD candidate studying plant–fungal interactions.

**Louis Kraft** is a masters student studying *de novo* transcriptome assembly.

**Venket Raghavan** and **Linda Rigerte** are scientific research assistants working on RNA-seq and plant–fungal interactions, respectively.
